# Optimal design of lattice structures for controllable extremal band gaps

**DOI:** 10.1038/s41598-019-46089-9

**Published:** 2019-07-10

**Authors:** Myung-Jin Choi, Myung-Hoon Oh, Bonyong Koo, Seonho Cho

**Affiliations:** 1 Department of Naval Architecture and Ocean Engineering, Seoul National University, 1 Gwanak-ro, Gwanak-gu, Seoul, 08826 Republic of Korea; 2 School of Mechanical Convergence System Engineering, Gunsan National University, Kunsan, 54150 Korea

**Keywords:** Mechanical engineering, Computational methods, Computational science

## Abstract

This paper presents very large complete band gaps at low audible frequency ranges tailored by gradient-based design optimizations of periodic two- and three-dimensional lattices. From the given various lattice topologies, we proceed to create and enlarge band gap properties through controlling neutral axis configuration and cross-section thickness of beam structures, while retaining the periodicity and size of the unit cell. Beam neutral axis configuration and cross-section thickness are parameterized by higher order B-spline basis functions within the isogeometric analysis framework, and controlled by an optimization algorithm using adjoint sensitivity. Our optimal curved designs show much more enhanced wave attenuation properties at audible low frequency region than previously reported straight or simple undulated geometries. Results of harmonic response analyses of beam structures consisting of a number of unit cells demonstrate the validity of the optimal designs. A plane wave propagation in infinite periodic lattice is analyzed within a unit cell using the Bloch periodic boundary condition.

## Introduction

Acoustic metamaterials with periodic arrangements of components have an important dynamic property of band gap that represents a certain frequency range where elastic wave or sound propagation through a material is prohibited. Due to its immense potential for novel applications like vibration and noise mitigations, and waveguides, there have been extensive research attentions to enhance the band gap property by engineering constituent material properties or structural geometries. The fundamental mechanism of band gap formations has been classified into the *Bragg scattering* and the *local resonance* of structural elements. In the Bragg-type one, destructive interferences of wave reflections due to structural periodicity account for the decays of waves at a certain frequency. However, since a wavelength in periodic lattices is scaled with unit cell size, the low frequency band gap requires a significant increase in overall structural dimension, which is impractical. Thus, for the band gaps of low frequency, locally resonating units have been successfully exploited to dissipate the energy of wave propagation around their resonance frequency. Liu *et al*.^1^ fabricated locally resonant structures, so-called sonic crystals, composed of hard spherical inclusion coated with a soft cladding and a stiff matrix, with much lower frequency of band gap formation than Bragg-type ones due to the localized vibrational motion of the inclusions. Bacigalupo *et al*.^2^ combined anti-chiral lattice structure with inertial resonators, and designed the number, arrangements, and material properties of the resonators to improve band gap properties using a nonlinear optimization algorithm. Matlack *et al*.^3^ embedded steel cubes as local resonators in a polycarbonate beam lattice, and altered the geometrical parameters of the lattice like the number of constituent beams, cross-section thickness of beams, unit cell size, and resonator filling fraction, which shows a variety of band gap formations due to different local resonant modes. Jensen^4^ studied in-plane wave propagation in 1-D and 2-D mass-spring models. This study showed that complete band gaps exist for a certain distribution of stiffness and mass, and demonstrated how band gaps can be created at low frequency ranges by introducing a local resonator into periodic structures. Martinsson and Movchan^5^ demonstrated that band gaps in lattice structures can be generated and controlled by introducing oscillators, and changing the added mass and the stiffness of supporting structures. Colquitt *et al*.^6^ demonstrated the filtration and focusing effects of elastic waves due to an elastic lens constructed by a diatomic lattice structure with non-uniformly distributed stiffness and mass. Instead of introducing local resonators, it is shown that single material systems could have band gaps by local resonances. Krödel *et al*.^7^ locally increased the wall thickness of hollow trusses, and showed that additional masses at truss junctions lead to broader band gaps at low frequencies due to the amplification of microscopic rotatory inertia. Wang *et al*.^8^ demonstrated band gap properties in beam lattices due to local resonances, and investigated the effects of lattice topologies and joint conditions, where the band gap properties are correlated with the average connectivity of a beam network, known as the coordination number. Warmuth *et al*.^9^ fabricated a three-dimensional cellular structure made of single phase titanium alloy having low frequency band gap using selected electron beam melting, and experimentally verified the band gap property. Li *et al*.^10^ attached cantilever beams to the structural frame to transfer elastic wave in the frame to the local resonance of attached beams, which consequently generates band gaps. Various lattice topologies of two-dimensional structures were suggested through parametric studies on configuration designs, including self-similar fractal^11^, star^12^, zig-zag^13^, and chiral^14^ shaped structures. Also, the introduction of undulated lattice geometries in the ligaments has been shown to generate band gap properties. Trainiti *et al*.^15^ showed that the wave propagation properties of lattice structures are significantly affected by the specific pattern of undulation due to the coupling of axial and flexural modes. Chen *et al*.^16^ also presented that the band gaps of low frequency can be generated within a square lattice by introducing sinusoidal undulations. There have also been studied for exploiting mathematical optimization methods to obtain the phononic crystal structures of large band gaps. Sigmund and Jensen^17^ performed a topology optimization, based on the solid isotropic material penalization (SIMP) method, to design periodic structures exhibiting band gap properties. Lu *et al*.^18^ synthesized a three-dimensional phononic crystal structure using the SIMP based topology optimization of two-phase material, where very large size of band gaps is generated at high frequency levels. Li *et al*.^19^ attained the band gaps of low frequency level by embedding inclusions in a base material through the bi-directional evolutionary structural optimization (BESO) process. In Wormser *et al*.^20^, a two-dimensional design obtained by the combination of shape and topology optimization is manually inserted into a three-dimensional structure having an enhanced band gap property. Diaz *et al*.^21^ studied the optimal mass distributions of plane grid structures to create and maximize band gaps, where the influence of skew angle of ligaments on band gap distribution was also identified.

Hughes *et al*.^22^ developed an isogeometric analysis (IGA) method that employs the same NURBS (non-uniform rational B-splines) basis functions as used in CAD description. The geometric properties of design are embedded into the NURBS basis functions and the control points whose perturbation naturally results in shape changes^23,24^. Thus, exact geometric models can be used in both response and shape sensitivity analyses, where normal vector and curvature are continuous over the whole design space, which leads to the enhanced shape sensitivity and consequently yields a precise optimal design. Choi and Cho^25^ synthesized a lattice structure of shape memory polymers possessing a target performance during shape recovery process, considering finite deformation. Choi *et al*.^26^ architected two- and three-dimensional lattice structures having a nearly constant extremal Poisson’s ratio −2 during finite deformation.

The previous studies for the analysis of band gap structures have a limitation that the layout of lattice is devised intuitively. Also, the sequel design optimization is performed only for topology optimization, which does not provide precise performances. This paper presents a systematic synthesis of unified isogeometric design for lattice structures achieving the enhanced band gap properties using a gradient-based optimization algorithm that considers both sizing and configuration design variables. We calculate wave dispersion relations in infinite periodic lattices using the Bloch theorem which reduces the maximization problem to that of a unit cell. Also, the IGA method is employed using the higher order B-spline basis functions for the spatial discretization of the given eigenvalue problem. This work paves the way to the systematic design of band gap structures possessing a designated performance in versatile engineering applications.

## Results and Discussions

The goal of the optimization is to maximize the relative band gap size between the two adjacent modes (*j*) and (*j* + 1). We pursue to maximize the lowest frequency of the overlying bands and minimize the maximum frequency of the underlying bands^17^. Denoting a set of configuration and sizing design variables as ${\bf{d}}\equiv \{{d}_{i}\}$, the optimization problem can be stated as: find **d** such that

1 $${\rm{Maximize}}\,f({\bf{d}})=2\frac{{{\rm{\min }}}_{{\bf{k}}}{\zeta }_{j+1}({\bf{k}},{\bf{d}})-{{\rm{\max }}}_{{\bf{k}}}{\zeta }_{j}({\bf{k}},{\bf{d}})}{{{\rm{\min }}}_{{\bf{k}}}{\zeta }_{j+1}({\bf{k}},{\bf{d}})+{{\rm{\max }}}_{{\bf{k}}}{\zeta }_{j}({\bf{k}},{\bf{d}})},\,\,{\bf{k}}\in {{\rm{\Gamma }}}_{IBZ},$$

2 $${\rm{subject}}\,{\rm{to}}\,\{\tilde{{\bf{K}}}({\boldsymbol{\mu }})-{\omega }^{2}\tilde{{\bf{M}}}({\boldsymbol{\mu }})\}\tilde{{\bf{u}}}={\bf{0}},\,\zeta \equiv {\omega }^{2},$$

3 $${g}_{k}({\bf{d}})\equiv \frac{1}{{L}_{k}({\bf{d}})}{\int }_{{{\rm{\Omega }}}_{k}}[{\{{\kappa }^{f}(\xi )/{\kappa }_{U}^{f}\}}^{2}-1]ds < 0,\,k=1,\,\ldots ,\,ne,$$

and

4 $${d}_{i}^{lower}\le {d}_{i}\le {d}_{i}^{upper},$$

where ${{\rm{\Gamma }}}_{IBZ}$, **k**, and ω represent the perimeter of irreducible Brillouin zone (IBZ), wave vector, and angular frequency, respectively. We define ${\boldsymbol{\mu }}\equiv \{{\mu }_{1},{\mu }_{2},{\mu }_{3}\}$ where ${\mu }_{i}\equiv {\bf{k}}\cdot {{\bf{b}}}_{i}\,(i=1,2,3)$ are the wave propagation constants. $\tilde{{\bf{K}}}$ and $\tilde{{\bf{M}}}$ denote the reduced stiffness and mass matrices, respectively. Eq. (3) represents the geometric constraints such that restricts the maximum curvature of ligaments to avoid the entanglement of ligaments due to abrupt design changes, and Ω_*k*_ represents a curve segment corresponding to *k*-th knot span among total *ne* knot spans, whose length is denoted by *L*_*k*_ (see Methods for more details on the NURBS curve geometry). Also, ${\kappa }^{f}$ and ${\kappa }_{U}^{f}$ respectively denote the Frenet-Serret curvature and its selected upper bound. Detailed expressions of calculating the curvature constraint and its design sensitivity was presented in reference^27^. In all the examples, the Modified Method of Feasible Directions (MMFD) algorithm is used in order to solve the nonlinear constrained optimization problems.

### Triangular lattice structure

**Figure 1:**
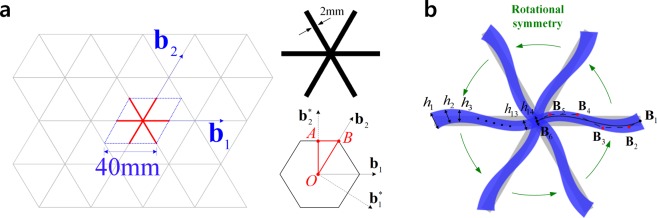
Triangular lattice structure. (**a**) Unit cell and irreducible first Brillouin zone. (**b**) Design parameterization.

A planar lattice structure composed of regular triangles whose side is 40 mm long, as illustrated in Fig. 1(a). Using the Bloch theorem in *Supplement A*, a unit cell can be defined by a translational periodicity along the directions of lattice base vectors **b**_1_ and **b**_2_. We select a rectangular cross-section with thickness *h*_0_ = 2 *mm* and depth *d*_0_ = 6 *mm*, and the material properties of Young’s modulus *E* = 1.14 *GPa*, Poisson’s ratio $\nu =0.3$, and density $\rho =1050\,kg/{m}^{3}$. The reciprocal lattice basis ${{\bf{b}}}_{1}^{\ast }$, ${{\bf{b}}}_{2}^{\ast }$, and the boundary of irreducible first Brillouin zone (O-A-B-O) are depicted in Fig. 1(a). The complete expressions of the lattice base vectors can be found in *Supplement B*. Figure 1(b) illustrates the design parameterization of configuration design in coarse level discretization by quartic B-spline basis function with 6 control points for each ligament. The *X*- and *Y*-directional changes of 4 control points depicted by red dots in Fig. 1(b) are selected as the configuration design variables. The end control point is fixed during the optimization process to maintain the translational periodicity and the unit cell size. The other five ligaments are designed by exploiting the rotational symmetry within the unit cell in the original design. Also, 14 thickness control coefficients corresponding to quartic B-spline basis functions are used as sizing design variables to parameterize cross-section thickness distribution within a ligament, i.e., *n*_*th*_ = 14 in Eq. (38).

**Figure 2:**
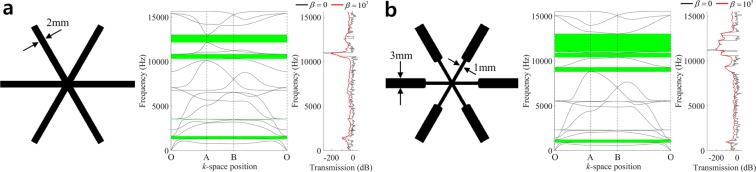
Comparison of band structures and frequency responses. (**a**) Original design, (**b**) Optimal design (Case #1).

Figure 2(a) shows that the triangular structure has four complete band gaps within the considered frequency range (0~15,655 Hz). Here and hereafter, we plot band diagrams with branches for the lowest 18 eigenmodes. Those band gaps are also identified from the drops in response illustrated in the wave transmission plot, where the undamped (*β* = 0) and damped (*β* = 10^3^) responses are compared, and *β* denotes the mass-proportional damping coefficient (see Methods and *Supplement E* for more details on the harmonic response analysis and the descriptions of finite structures, respectively). In the middle of the fourth band gap frequency range, a response peak is observed for the undamped case (*β* = 0; black line). This can be attributed to a resonance effect due to the reflective waves from the boundaries^17^. When we consider a structural damping (*β* = 10^3^; red line), the resonance peak disappears and relatively low transmission is apparently shown in the frequency range of the fourth band gap. Starting from the straight geometry in Fig. 2(a), we perform a design optimization to maximize the first band gap between 3^rd^-4^th^ modes (Case #1). We obtain the optimal design of Fig. 2(b) where the geometry of neutral axis does not change but the cross-section of ligament becomes thinner around the junction and thicker in the rest of ligaments. The target band gap between 3^rd^-4^th^ modes is located at lower frequency region than that of the original design due to the decrease of stiffness near the junction, and the band gap size slightly increases from 307 to 318 Hz. Interestingly, in the optimal design, a large band gap appears between 15^th^ and 16^th^ modes in the optimal design, and two nearly flat branches between the third and fourth band gaps of the optimal design stand for nearly zero group velocity in all the wave propagation directions, which represent isolated (standing) waves. We also observe that the damped response in the plot of transmission is much smoother than the undamped case, and the response drop in the fourth band gap frequency range is more apparently observed as the local resonance peak disappears. Table 1 compares the band gap frequencies of the original and the optimal designs.

**Table 1:** Comparison of sizes and frequency ranges.

	Band gap #	Band gap size (Hz)	Lower bound of band gap	
			Mode #	Frequency (Hz)
Original design	1	307.0	3	1316.6
	2	31.5	6	3521.9
	3	533.0	12	10325.9
	4	862.0	14	12162.7
Optimal design (case #1)	1	318.0	3	916.9
	2	496.2	12	8812.6
	3	510.2	13	10400.7
	4	2010.9	15	11029.3

**Figure 3:**
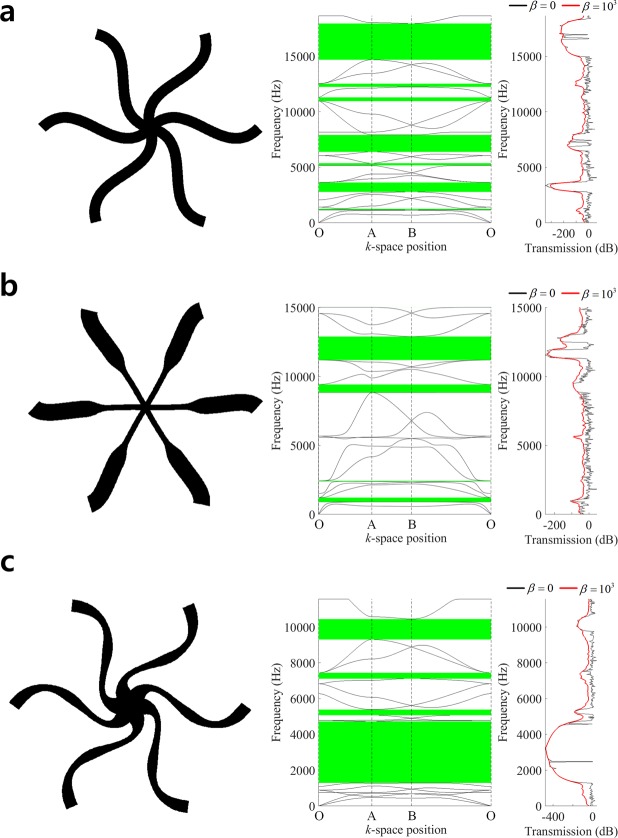
Comparison of band structures and frequency responses. (**a**) Undulated design result, (**b**) Optimal design result (Case #2), (**c**) Optimal design result (Case #3).

An undulated design of Fig. 3(a) is generated by perturbing the first four design variables such that *d*_1_ = *d*_2_ = −2.5 *mm* and *d*_3_ = *d*_4_ = 2.5 *mm*, which turns out to have more band gaps, compared with the original straight design, as shown in Fig. 3(a). We perform design optimizations with this original undulated design for two cases of target band gaps; a band gap between 3^rd^ and 4^th^ modes (Case #2) and a band gap between 6^th^ and 7^th^ modes (Case #3). The obtained optimal designs and band diagrams for the two cases are shown in Fig. 3(b,c). Table 2 lists the sizes and frequency ranges of their band gaps. In the optimal design of case #2, the cross-section of ligament becomes thinner around the junction and thicker in the rest of ligaments, which decreases overall stiffness and consequently the first band gap has a lower frequency range than that of the original undulated design (from 1,156 to 908 Hz). The band gap size increases from 113 to 313 Hz. In the optimal design of case #3, the band gap between 3^rd^ and 4^th^ modes is suppressed but very large band gap appears at the low frequency range of 1,290~4,724 Hz. In the band diagram of Fig. 3(c), the first and second band gaps are nearly unified into a single band gap, since the branches for the 7^th^–9^th^ modes are nearly flat and almost coincide at the frequency range around 4,471 Hz. In the plots of wave transmission of Fig. 3, by introducing the structural damping, local resonance peaks in the band gap ranges are removed, and the band gaps are more clearly identified by the transmission drops.

**Table 2:** Comparison of band gap sizes and frequency ranges.

	Band gap #	Band gap size (Hz)	Lower bound of band gap	
			Mode#	Frequency (Hz)
Original undulated design	1	113.1	3	1156.0
	2	841.3	6	2789.4
	3	196.1	9	5168.1
	4	1513.9	11	6402.3
	5	337.5	14	10972.9
	6	301.1	15	12243.6
	7	3263.7	17	14693.8
Optimal design (case #2)	1	312.9	3	908.0
	2	46.6	6	2393.4
	3	582.6	9	8837.6
	4	1705.4	12	11192.2
Optimal design (case #3)	1	3181.2	6	1290.1
	2	252.7	9	4471.3
	3	311.0	12	5078.8
	4	321.9	15	7117.5
	5	1142.2	17	9304.7

### Hexagonal honeycomb

**Figure 4:**
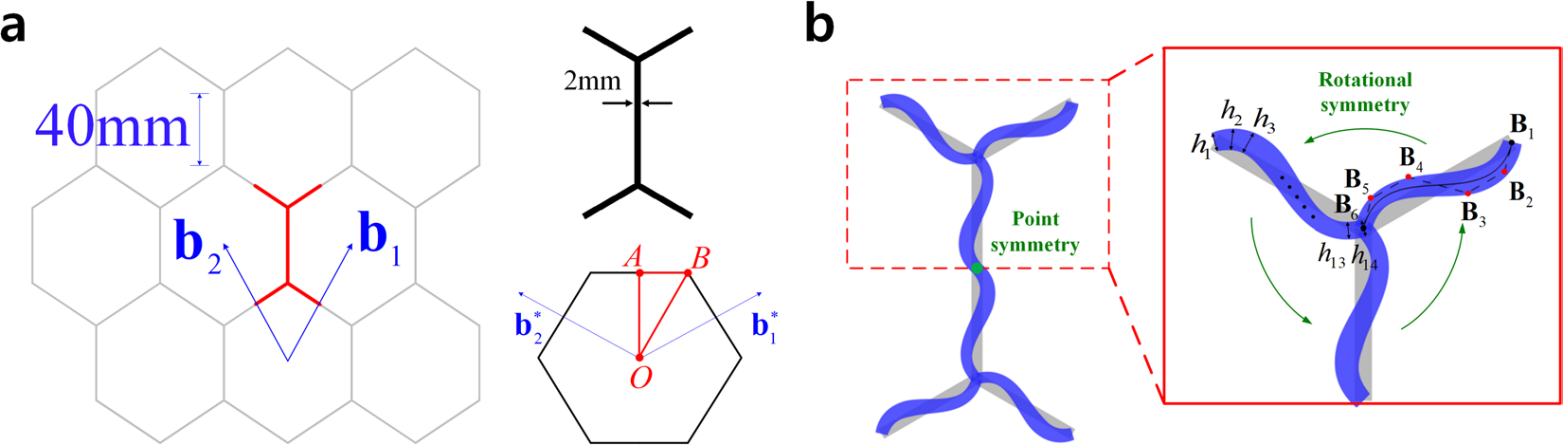
Hexagonal honeycomb structure. (**a**) Unit cell and irreducible first Brillouin zone, (**b**) Design parameterization.

We consider a planar hexagonal honeycomb structure illustrated in Fig. 4(a). A minimal repeated unit, which is arranged in infinite lattice structures by translations in the directions of **b**_1_ and **b**_2_, has a rectangular cross-section with uniform thickness *h*_0_ = 2 *mm* and depth *d*_0_ = 6 *mm*. The corresponding reciprocal base vectors and irreducible Brillouin zone is obtained as shown in Fig. 4(a). Material properties of Young’s modulus $E=1.14\,GPa$, Poisson’s ratio $\nu =0.3$, and density $\rho =1050\,kg/{m}^{3}$ are selected. A single ligament configuration within the half part of the unit cell is parameterized by 8 configuration design variables which are X- and Y-directional position changes of four control points depicted by red dots in Fig. 4(b). The other ligaments are parameterized by using a rotational symmetry within the half part and a point symmetry within the unit cell. Quartic B-spline basis functions are used. Also, 14 control coefficients are used to represent the continuous thickness of cross-section, i.e., *n*_*th*_ = 14.

**Figure 5:**
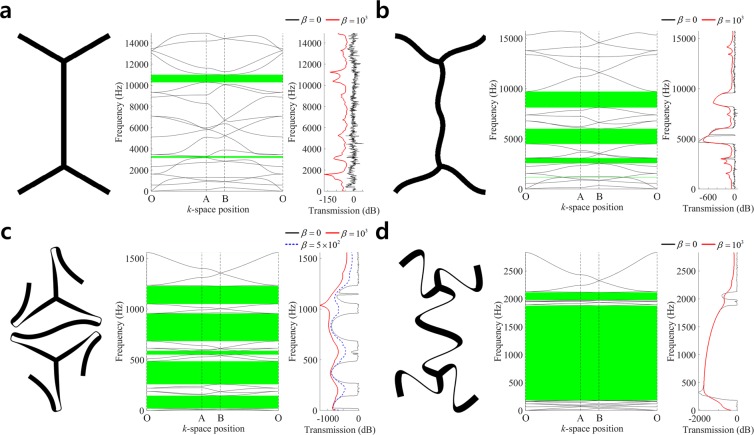
Comparison of band structures and frequency responses. (**a**) Straight design, (**b**) Undulated design, (**c**) Optimal undulated design (Case #4), (**d**) Optimal undulated design (Case #5).

Figure 5 shows the comparison of band diagrams and wave transmission plots for the various designs. A couple of complete band gaps are observed in the band diagram of Fig. 5(a), although the wave transmission of the undamped case (*β* = 0; black line) does not show distinctive transmission drops in those frequency ranges. The structural damping diminishes the reflective waves from the boundaries, so that it apparently identifies low transmissions in the band gap intervals (*β* = 10^3^; red line). The first very low transmission around 1,600 Hz identifies the partial band gap between the 4^th^ and 5^th^ modes. An undulated design of Fig. 5(b) is introduced by perturbing design variables as *d*_1_ = *d*_2_ = 1.25 *mm* and *d*_3_ = *d*_4_ = −1.25 *mm*, and it has four complete band gaps. We select two target band gaps; the band gap between 3^rd^ and 4^th^ modes (Case #4) which initially appear very thin and the band gap between 9^th^ and 10^th^ modes (Case #5). In the case #4 of optimal undulated design, the target band gap between 3^rd^ and 4^th^ modes is significantly enlarged and located at lower frequency level of the region 20~146 Hz. Also, several other band gaps show very low wave transmissions. In the case #5 of optimal undulated design, a large complete band gap is generated between 9^th^ and 10^th^ modes in the frequency region 182~1,878 Hz, and the wave transmission is much lower than that of the original undulated design. The wave transmissions considering damping in Fig. 5(b–d) show much smoother responses than the undamped responses, and are free-from local peaks in band gap ranges. It is also observed in Fig. 5(c) that the weaker damping (*β* = 5 × 10^2^; blue dashed line) gives more distinctive response drops in band gap ranges. Table 3 compares the sizes and frequency regions of band gaps in the original straight design, original undulated design, and two optimal designs.

**Table 3:** Comparison of band gap sizes and frequency ranges in hexagonal honeycomb lattice.

	Band gap #	Band gap size (Hz)	Lower bound of band gap	
			Mode #	Frequency (Hz)
Original design	1	151.6	6	3162.1
	2	719.2	14	10281.7
Original undulated design	1	29.6	3	1165.9
	2	501.7	6	2582.4
	3	1513.7	9	4489.5
	4	1597.8	13	8114.7
Optimal design (case #4)	1	125.8	3	20.4
	2	227.4	7	258.2
	3	38.2	9	550.9
	4	272.2	12	683.9
	5	177.5	15	1050.1
Optimal design (case #5)	1	1695.9	9	182.4
	2	136.1	15	1981.0

### Three-dimensional simple cubic structure

**Figure 6:**
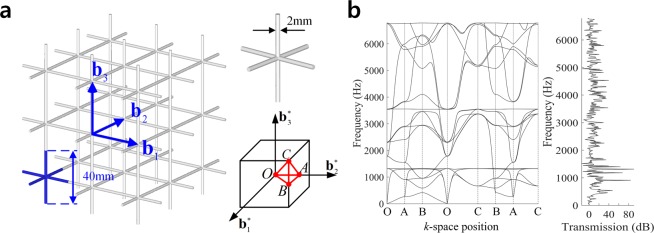
Simple cubic lattice structure. (**a**) Straight design, (**b**) Band structure and frequency response.

A three-dimensional cubic lattice structure is shown in Fig. 6(a). Using the translational periodicity in the direction of bases **b**_1_, **b**_2_, and **b**_3_, a unit cell is defined. We select a circular cross-section with a uniform diameter of *d*_0_ = 2 *mm*. Material properties of Young’s modulus $E=1.14\,GPa$, Poisson’s ratio $\nu =0.3$, and mass density $\rho =1050\,kg/{m}^{3}$ are selected. Figure 6(a) shows the reciprocal lattice bases ${{\bf{b}}}_{1}^{\ast }$, ${{\bf{b}}}_{2}^{\ast }$, and ${{\bf{b}}}_{3}^{\ast }$ whose detailed expressions are found in *Supplement B*. A perimeter of irreducible Brillouin zone is indicated by the red lines. Figure 6(b) shows the band diagram and wave transmission, where no complete band gap is generated.

**Figure 7:**
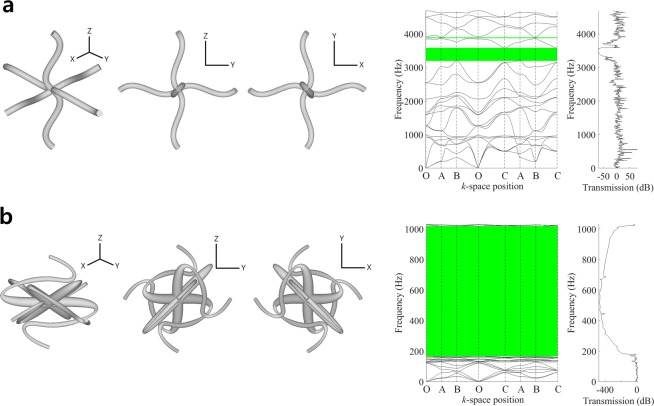
Comparison of band structures and frequency responses. (**a**) Original undulated design, (**b**) Optimal undulated design.

A single ligament configuration is parameterized by 8 configuration design variables. 24 thickness control coefficients are used. We introduce a geometric undulation of ligaments as illustrated in Fig. 7(a), then the undulated design shows a couple of complete band gaps. The first band gap between 15^th^ and 16^th^ modes apparently shows low wave transmissions. From this undulated design, we perform design optimization to maximize the first band gap (Case #6), and finally obtain the optimal design shown in Fig. 7(b), where the optimal design attained significantly larger band gap in the frequency range of 166~1,019 Hz with enhanced wave attenuation, compared with those of original undulated design. Table 4 compares the sizes and frequency regions of band gaps in the original undulated and optimal designs.

**Table 4:** Comparison of band gap sizes and frequency ranges in simple cubic lattice.

	Band gap #	Band gap size	Lower bound of band gap	
		(Hz)	Mode #	Frequency (Hz)
Original undulated design	1	387.9	15	3198.9
	2	24.9	16	3875.1
Optimal design	1	853.7	15	165.6

For more design cases, the readers refer to *Supplement C* (square lattice) and *Supplement D* (Kagomé lattice). The histories of design optimization for the cases are found in *Supplement F*.

## Methods

### Isogeometric analysis of elastic wave propagation in shear-deformable beam structures

We briefly introduce the construction of NURBS basis, and a representation of NURBS curve. Also, we explain the isogeometric analysis of elastic wave propagation in shear-deformable (Timoshenko) beam structures.

#### NURBS curves

The geometry of neutral axis and the distribution of cross-sectional thickness are expressed by NURBS basis functions constructed from B-splines. A set of knots in one dimension consists of coordinates in parametric space, denoted by

5 $${\boldsymbol{\xi }}=\{{\xi }_{1},{\xi }_{2},\cdots ,{\xi }_{n+p+1}\},$$

where *p* and *n* are respectively the order of basis function, and the number of control points. We term an interval between two distinct consecutive knots as a *knot span*, which subdivides a NURBS curve into curve segments, *i.e*., elements. The B-spline basis functions are obtained, in a recursive manner, as

6 $${N}_{I}^{0}(\xi )=\{\begin{array}{ll}1 & {\rm{if}}\,{\xi }_{I}\le \xi  < {\xi }_{I+1}\\ 0 & {\rm{otherwise}}\end{array},\,(p=0)$$

and

7 $${N}_{I}^{p}(\xi )=\frac{\xi -{\xi }_{I}}{{\xi }_{I+p}-{\xi }_{I}}{N}_{I}^{p-1}(\xi )+\frac{{\xi }_{I+p+1}-\xi }{{\xi }_{I+p+1}-{\xi }_{I+1}}{N}_{I+1}^{p-1}(\xi ),\,(p=1,2,3,\ldots ),$$

From the B-spline basis ${N}_{I}^{p}(\xi )$ and the weight *w*_*I*_, NURBS basis function ${W}_{I}(\xi )$ is defined by

8 $${W}_{I}(\xi )\equiv \frac{{N}_{I}^{p}(\xi ){w}_{I}}{{\sum }_{J=1}^{n}{N}_{J}^{p}(\xi ){w}_{J}}.$$

The NURBS basis function of order *p* has *C*^*p*-*k*^ continuity at a knot multiplicity *k*. A geometric point **x** on a beam neutral axis is expressed, in terms of NURBS basis function ${W}_{I}(\xi )$ and control point **B**_*N*_, as

9 $${\bf{x}}(\xi )={\sum }_{N=1}^{n}{W}_{N}(\xi ){{\bf{B}}}_{N},$$

where *n* denotes the number of control points, and the variation of control point **B**_*N*_ results in configuration changes.

#### Linear kinematics and governing equations

We consider structural dynamics with infinitesimal amplitudes, where a linearized kinematics consistently derived from a general nonlinear formulation^28^ is utilized. A neutral axis of beams is parameterized using an arc-length coordinate *s*. A cross-sectional orientation is defined by an orthonormal frame with base vectors ${{\bf{j}}}_{1}(s),{{\bf{j}}}_{2}(s),{{\bf{j}}}_{3}(s)\in {{\bf{R}}}^{3}$, where the unit tangent vector is obtained by ${{\bf{j}}}_{1}(s)\equiv {{\boldsymbol{\phi }}}_{0,s}$ due to the arc-length parameterization, and assumed to be orthogonal to the cross-section. ${(\cdot )}_{,s}$ denotes the partial differentiation with respect to the arc-length parameter. We also define the global Cartesian base vectors by ${{\bf{e}}}_{1},{{\bf{e}}}_{2},{{\bf{e}}}_{3}\in {{\bf{R}}}^{3}$. The linearized (material form) axial-shear and bending-torsional strain measures derived in the reference^29^ are respectively evaluated at an undeformed state, which result in

10 $${\boldsymbol{\Gamma }}(s,t)\equiv {{\boldsymbol{\Lambda }}}_{0}{(s)}^{T}\{\frac{\partial }{\partial s}{{\bf{z}}}_{t}+{{\bf{j}}}_{1}(s)\times {{\boldsymbol{\theta }}}_{t}\},$$

and

11 $${\boldsymbol{\Omega }}(s,t)\equiv {{\boldsymbol{\Lambda }}}_{0}{(s)}^{T}\frac{\partial {{\boldsymbol{\theta }}}_{t}}{\partial s}.$$

${{\bf{z}}}_{t}\equiv {\bf{z}}(s,t)$ denotes the displacement, and ${{\boldsymbol{\theta }}}_{t}\equiv {\boldsymbol{\theta }}(s,t)$ denote the infinitesimal rotation vector that describes the motion of rigid cross-section. ${{\boldsymbol{\Lambda }}}_{0}(s)\equiv [{{\bf{j}}}_{1}(s),{{\bf{j}}}_{2}(s),{{\bf{j}}}_{3}(s)]$ defines the rotational transformation matrix such that

12 $${{\bf{j}}}_{I}(s)={{\boldsymbol{\Lambda }}}_{0}(s){{\bf{e}}}_{I},\,I=1,\,2,\,3.$$

An elastic strain energy is defined by

13 $$U\equiv \frac{1}{2}{\int }_{{\rm{\Omega }}}\{{\boldsymbol{\Gamma }}(s,t)\cdot {\bf{N}}(s,t)+{\boldsymbol{\Omega }}(s,t)\cdot {\bf{M}}(s,t)\}ds,$$

where **N**(*s*, *t*) and **M**(*s*, *t*) are the material form resultant force and moment over the cross-section, which are related to spatial forms through

14 $$\begin{array}{c}{\bf{n}}(s,t)={{\boldsymbol{\Lambda }}}_{0}(s){\bf{N}}(s,t)\\ {\bf{m}}(s,t)={{\boldsymbol{\Lambda }}}_{0}(s){\bf{M}}(s,t)\end{array}\}.$$

A linear elastic constitutive relation is given by

15 $$\begin{array}{l}{\bf{N}}(s,t)={{\bf{C}}}_{F}{\boldsymbol{\Gamma }}(s,t)\\ {\bf{M}}(s,t)={{\bf{C}}}_{M}{\boldsymbol{\Omega }}(s,t)\end{array}\},$$

where the constitutive matrices ${{\bf{C}}}_{F}$ and ${{\bf{C}}}_{M}$ are defined by

16 $$\begin{array}{ccc}{{\bf{C}}}_{F} & = & diag[EA,G{A}_{2},G{A}_{3}]\\ {{\bf{C}}}_{M} & = & diag[G{I}_{p},E{I}_{2},E{I}_{3}]\end{array}\}.$$

*diag*[*a*, *b*, *c*] represents a diagonal matrix of components *a*, *b*, and *c*. *A* represents the cross-sectional area, and ${A}_{2}\equiv {k}_{2}A$, ${A}_{3}\equiv {k}_{3}A$ where *k*_2_ and *k*_3_ denote the shear correction factors. *I*_*p*_ denotes the polar moment of inertia, and *I*_2_ and *I*_3_ mean two principal moments of inertia. *E* and *G* denote Young’s modulus and shear modulus, respectively. We also define a kinetic energy by

17 $$T\equiv \frac{1}{2}{\int }_{{\rm{\Omega }}}\{\rho A{{\bf{z}}}_{t,t}\cdot {{\bf{z}}}_{t,t}+{{\boldsymbol{\theta }}}_{t,t}\cdot ({{\bf{I}}}_{\rho }{{\boldsymbol{\theta }}}_{t,t})\}ds,$$

where ${(\cdot )}_{,t}$ denotes the partial differentiation with respect to the time. *ρ* and ${{\bf{I}}}_{\rho }\equiv \rho \cdot diag[{I}_{p},{I}_{2},{I}_{3}]$ respectively denote the mass density and inertia tensor. A work done by external loads is expressed as

18 $$W\equiv {\int }_{{\rm{\Omega }}}\{{{\bf{n}}}_{ext}(s)\cdot {\bf{z}}(s,t)+{{\bf{m}}}_{ext}(s)\cdot {\boldsymbol{\theta }}(s,t)\}ds,$$

where **n**_*ext*_(*s*) and **m**_*ext*_(*s*) denotes the distributed external force and moment, respectively. To derive equilibrium equations from time *t*_1_ to time *t*_2_, we employ the Hamilton’s principle such that^30^

19 $$\delta \,{\int }_{{{\rm{t}}}_{1}}^{{t}_{2}}(U-T-W)dt=0,$$

where $\delta (\,\cdot \,)$ defines the first variation. Substituting Eqs. (13), (17), and (18) into Eq. (19), and applying the integration by parts and homogeneous boundary conditions lead to the following linear and angular momentum balance equations

20 $$\begin{array}{rcl}{{\bf{n}}}_{t,s}+{{\bf{n}}}_{ext} & = & \rho A{{\bf{z}}}_{t,tt}\\ {{\bf{m}}}_{t,s}+{{\bf{j}}}_{1}\times {{\bf{n}}}_{t}+{{\bf{m}}}_{ext} & = & {{\bf{I}}}_{\rho }{{\boldsymbol{\theta }}}_{t,tt}\end{array}\},$$

where ${{\bf{n}}}_{t}\equiv {\bf{n}}(s,t)$, ${{\bf{m}}}_{t}\equiv {\bf{m}}(s,t)$, and ${(\cdot )}_{,tt}$ denotes the second-order partial derivative with respect to the time. Assuming time-harmonic solutions, we have the following.

21 $$\begin{array}{rcl}{\bf{z}}(s,t) & = & {\bf{z}}(s){e}^{-i\omega t}\\ {\boldsymbol{\theta }}(s,t) & = & {\boldsymbol{\theta }}(s){e}^{-i\omega t}\end{array}\},$$

where *ω* denotes the angular frequency. In the IGA method, the linear and angular displacements are discretized using the same NURBS basis function ${W}_{N}(\xi )$ used to express the geometry in Eq. (9) and the response coefficients **y**_*N*_ and **θ**_*N*_ as

22 $$\begin{array}{rcl}{\bf{z}}(s(\xi )) & = & {\sum }_{N=1}^{n}{W}_{N}(\xi ){{\bf{y}}}_{N}\\ {\boldsymbol{\theta }}(s(\xi )) & = & {\sum }_{N=1}^{n}{W}_{N}(\xi ){{\boldsymbol{\theta }}}_{N}\end{array}\}.$$

It is noted that, hereafter, the argument $s(\xi )$ is often omitted for brevity. The linearized translational and rotational strains are discretized as

23 $$\begin{array}{rcl}{\boldsymbol{\Gamma }} & = & {{\boldsymbol{\Lambda }}}_{0}^{T}{\sum }_{N=1}^{n}({W}_{N,s}{{\bf{y}}}_{N}+{{\bf{j}}}_{1}\times {W}_{N}{{\boldsymbol{\theta }}}_{N})\\ {\boldsymbol{\Omega }} & = & {{\boldsymbol{\Lambda }}}_{0}^{T}{\sum }_{N=1}^{n}{W}_{N,s}{{\boldsymbol{\theta }}}_{N}\end{array}\},$$

where *W*_*N,S*_ defines the differentiation of NURBS basis function with respect to the arc-length coordinate, calculated by using a chain rule of differentiation^31^. From the governing equations of Eq. (20), using the principle of virtual work and substituting Eq. (21), we have the following variational equation for response ${\boldsymbol{\eta }}\equiv ({\bf{z}},{\boldsymbol{\theta }})$.

24 $$a({\boldsymbol{\eta }},\bar{{\boldsymbol{\eta }}})={\omega }^{2}d({\boldsymbol{\eta }},\bar{{\boldsymbol{\eta }}}),\,\forall \bar{{\boldsymbol{\eta }}}\in \bar{Z},$$

where $\bar{Z}$ defines the space of kinematically admissible virtual response $\bar{{\boldsymbol{\eta }}}\equiv (\bar{{\bf{z}}},\bar{{\boldsymbol{\theta }}})$, and the strain and kinetic energy bilinear forms are obtained by

25 $$a({\boldsymbol{\eta }},\bar{{\boldsymbol{\eta }}})\equiv {\int }_{{\rm{\Omega }}}({\bar{{\boldsymbol{\Gamma }}}}^{T}{{\bf{C}}}_{F}{\boldsymbol{\Gamma }}+{\bar{{\boldsymbol{\Omega }}}}^{T}{{\bf{C}}}_{M}{\boldsymbol{\Omega }})ds,$$

and

26 $$d({\boldsymbol{\eta }},\bar{{\boldsymbol{\eta }}})\equiv {\int }_{{\rm{\Omega }}}({\bar{{\bf{z}}}}^{T}\rho A{\bf{z}}+{\bar{{\boldsymbol{\theta }}}}^{T}{{\bf{I}}}_{\rho }{\boldsymbol{\theta }})ds.$$

$\overline{(\,\cdot \,)}\equiv \delta (\,\cdot \,)$ denotes the first variation or the virtual quantity, that is, $\bar{{\boldsymbol{\Gamma }}}\equiv {\boldsymbol{\Gamma }}(\bar{{\bf{z}}},\bar{{\boldsymbol{\theta }}})$ and $\bar{{\boldsymbol{\Omega }}}\equiv {\boldsymbol{\Omega }}(\bar{{\boldsymbol{\theta }}})$. From Eq. (24), using Eqs (22) and (23), we have the following discretized form of a generalized eigenvalue problem.

27 $$({\bf{K}}-{\omega }^{2}{\bf{M}}){\bf{u}}={\bf{0}},$$

where **K** and **M** respectively denote the assembled global stiffness and mass matrices, and **u** is a global assembly of response coefficient vectors **y**_*N*_ and **θ**_*N*_.

#### Bloch periodic boundary condition

From the Bloch periodic condition, we have the following transformation.

28 $${\bf{u}}={\bf{T}}({\boldsymbol{\mu }})\tilde{{\bf{u}}},$$

where $\tilde{{\bf{u}}}$ denotes a global assembly of reduced response coefficients. Substituting Eq. (28) into Eq. (27) and pre-multiplying the resulting equation with **T**(**μ**)^*H*^ yields the following generalized Hermitian eigenvalue problem within a unit cell^32,33^

29 $$\{\tilde{{\bf{K}}}({\boldsymbol{\mu }})-{\omega }^{2}\tilde{{\bf{M}}}({\boldsymbol{\mu }})\}\tilde{{\bf{u}}}={\bf{0}},$$

where $\tilde{{\bf{K}}}({\boldsymbol{\mu }})\equiv {\bf{T}}{({\boldsymbol{\mu }})}^{H}{\bf{K}}{\bf{T}}({\boldsymbol{\mu }})$ and $\tilde{{\bf{M}}}({\boldsymbol{\mu }})\equiv {\bf{T}}{({\boldsymbol{\mu }})}^{H}{\bf{M}}{\bf{T}}({\boldsymbol{\mu }})$ defines the reduced stiffness and mass matrices, and ${(\cdot )}^{H}$ denotes the conjugate transpose. Eq. (29) gives the eigenvalues $\zeta \equiv {\omega }^{2}$ for a given propagation constants ${\boldsymbol{\mu }}\equiv \{{\mu }_{1},{\mu }_{2},{\mu }_{3}\}$.

### Adjoint design sensitivity analysis

#### Configuration design sensitivity analysis

A configuration design velocity field implies a mapping rate between the original design and the perturbed one, and is given by a linear combination of NURBS basis function and the perturbation of control points as

30 $${\bf{V}}={\sum }_{N=1}^{n}{W}_{N}(\xi )\delta {{\bf{B}}}_{N}.$$

For a given propagation constant ${\boldsymbol{\mu }}\in {{\rm{\Gamma }}}_{IBZ}$, Eq. (24) can be rewritten for a response ${\boldsymbol{\eta }}\equiv ({\bf{z}},{\boldsymbol{\theta }})$ within a unit cell satisfying the Bloch periodic boundary condition, as

31 $$a({\boldsymbol{\mu }};{\boldsymbol{\eta }},\bar{{\boldsymbol{\eta }}})=\zeta d({\boldsymbol{\mu }};{\boldsymbol{\eta }},\bar{{\boldsymbol{\eta }}}),\,\,{\rm{\forall }}\bar{{\boldsymbol{\eta }}}\in {\mathbb{Z}},$$

where $\zeta \equiv {\omega }^{2}$ and $\bar{{\mathbb{Z}}}$ defines the complex space of kinematically admissible virtual responses. ${{\rm{\Gamma }}}_{IBZ}$ denotes a perimeter of irreducible Brillouin zone (IBZ). Assuming that the translational periodicity and unit cell dimension do not have design dependences, taking the material derivative of both sides of Eq. (31) and rearranging. terms gives the following.

32 $$\begin{array}{rcl}a({\boldsymbol{\mu }};\dot{{\boldsymbol{\eta }}},\bar{{\boldsymbol{\eta }}})+{a^{\prime} }_{V}({\boldsymbol{\mu }};{\boldsymbol{\eta }},\bar{{\boldsymbol{\eta }}}) & = & \zeta \{d({\boldsymbol{\mu }};\dot{{\boldsymbol{\eta }}},\bar{{\boldsymbol{\eta }}})+{d^{\prime} }_{V}({\boldsymbol{\mu }};{\boldsymbol{\eta }},\bar{{\boldsymbol{\eta }}})\}\\  &  & +\,\dot{\zeta }d({\boldsymbol{\mu }};{\boldsymbol{\eta }},\bar{{\boldsymbol{\eta }}}),\,\forall \bar{{\boldsymbol{\eta }}}\in \bar{{\mathbb{Z}}},\end{array}$$

where $(\,\mathop{{\boldsymbol{\cdot }}}\limits^{\cdot }\,)$ denotes a material derivative or configuration design sensitivity, and sometimes we denote the material derivative by a superscript dot ${(\cdot )}^{\cdot }$ for convenience. Evaluating Eq. (32) at $\bar{{\boldsymbol{\eta }}}={\boldsymbol{\eta }}$ since they belong to the same function space $\bar{{\mathbb{Z}}}$ and using the normalization condition of $d({\boldsymbol{\mu }};{\boldsymbol{\eta }},{\boldsymbol{\eta }})=1$, we have the configuration design sensitivity expression for eigenvalues.

33 $$\dot{\zeta }={a^{\prime} }_{V}({\boldsymbol{\mu }};{\boldsymbol{\eta }},{\boldsymbol{\eta }})-\zeta {d^{\prime} }_{V}({\boldsymbol{\mu }};{\boldsymbol{\eta }},{\boldsymbol{\eta }}).$$

The explicit design dependence terms of strain and kinetic energy forms are given by

34 $${a^{\prime} }_{V}({\boldsymbol{\mu }};{\boldsymbol{\eta }},{\boldsymbol{\eta }})\equiv {\int }_{{\rm{\Omega }}}[\begin{array}{c}{{\boldsymbol{\Gamma }}{\boldsymbol{^{\prime} }}}_{V}{({\boldsymbol{\eta }})}^{H}{{\bf{C}}}_{F}{\boldsymbol{\Gamma }}({\boldsymbol{\eta }})+{{\boldsymbol{\Omega }}{\boldsymbol{^{\prime} }}}_{V}{({\boldsymbol{\eta }})}^{H}{{\bf{C}}}_{M}{\boldsymbol{\Omega }}({\boldsymbol{\eta }})+{\boldsymbol{\Gamma }}{({\boldsymbol{\eta }})}^{H}{{\bf{C}}}_{F}{{\boldsymbol{\Gamma }}{\boldsymbol{^{\prime} }}}_{V}({\boldsymbol{\eta }})\\ +{\boldsymbol{\Omega }}{({\boldsymbol{\eta }})}^{H}{{\bf{C}}}_{M}{{\boldsymbol{\Omega }}{\boldsymbol{^{\prime} }}}_{V}({\boldsymbol{\eta }})+\{{\boldsymbol{\Gamma }}{({\boldsymbol{\eta }})}^{H}{{\bf{C}}}_{F}{\boldsymbol{\Gamma }}({\boldsymbol{\eta }})+{\boldsymbol{\Omega }}{({\boldsymbol{\eta }})}^{H}{{\bf{C}}}_{M}{\boldsymbol{\Omega }}({\boldsymbol{\eta }})\}{\nabla }_{s}\cdot {\bf{V}}\end{array}]ds$$

and

35 $${d^{\prime} }_{V}({\boldsymbol{\mu }};{\boldsymbol{\eta }},{\boldsymbol{\eta }})\equiv {\int }_{{\rm{\Omega }}}({{\bf{z}}}^{H}{A}_{\rho }{\bf{z}}+{{\boldsymbol{\theta }}}^{H}{{\bf{I}}}_{\rho }{\boldsymbol{\theta }}){\nabla }_{s}\cdot {\bf{V}}ds,$$

where ${\nabla }_{s}\cdot {\bf{V}}\equiv {{\bf{V}}}_{,s}\cdot {{\bf{j}}}_{1}$ and the following are used.

36 $${\{{\boldsymbol{\Gamma }}({\boldsymbol{\eta }})\}}^{\cdot }={{\boldsymbol{\Lambda }}}_{0}^{T}({\dot{{\bf{z}}}}_{,s}-\dot{{\boldsymbol{\theta }}}\times {{\bf{j}}}_{1})+\{{{\boldsymbol{\Lambda }}}_{0}^{T}(\,-\,{{\bf{z}}}_{,s}{{\rm{\nabla }}}_{s}\cdot {\bf{V}}-{\boldsymbol{\theta }}\times {{\bf{j}}}_{1}^{\cdot })+{{\dot{{\boldsymbol{\Lambda }}}}_{0}}^{T}{{\boldsymbol{\Lambda }}}_{0}{\boldsymbol{\Gamma }}\}\equiv {\boldsymbol{\Gamma }}(\mathop{{\boldsymbol{\eta }}}\limits^{\cdot })+{{\boldsymbol{\Gamma }}{\rm{^{\prime} }}}_{V}({\boldsymbol{\eta }}),$$

and

37 $${\{{\boldsymbol{\Omega }}({\boldsymbol{\eta }})\}}^{\cdot }={{\boldsymbol{\Lambda }}}_{0}^{T}{\mathop{{\boldsymbol{\theta }}}\limits^{\cdot }}_{,s}+\{{{\boldsymbol{\Lambda }}}_{0}^{T}(-\,{{\boldsymbol{\theta }}}_{,s}{{\rm{\nabla }}}_{s}\cdot {\bf{V}})+{{\mathop{{\boldsymbol{\Lambda }}}\limits^{\cdot }}_{0}}^{T}{{\boldsymbol{\Lambda }}}_{0}{\boldsymbol{\Omega }}\}\equiv {\boldsymbol{\Omega }}(\dot{{\boldsymbol{\eta }}})+{{\boldsymbol{\Omega }}{\boldsymbol{^{\prime} }}}_{V}({\boldsymbol{\eta }}).$$

Compared with the finite element sensitivity, the isogeometric approach prevents the loss of higher order geometric information so that designers can obtain more precise configuration design sensitivity, which leads to a precise result of configuration design optimization^23^.

#### Sizing design sensitivity analysis

The cross-section thickness distributions along beam members can be continuously parameterized by combining thickness coefficients assigned to control points and the NURBS basis functions, as^34^

38 $$h={\sum }_{I=1}^{{n}_{th}}{h}_{I}{W}_{I}(\xi ),$$

where *h*_*I*_ denotes the *I*-th thickness control coefficient, and *n*_*th*_ denotes the number of thickness control coefficients in each patch. The sizing design sensitivity can be evaluated by

39 $$\zeta ^{\prime} ={a^{\prime} }_{\delta {\bf{u}}}({\boldsymbol{\mu }};{\boldsymbol{\eta }},{\boldsymbol{\eta }})-\zeta {d^{\prime} }_{\delta {\bf{u}}}({\boldsymbol{\mu }};{\boldsymbol{\eta }},{\boldsymbol{\eta }}),$$

where $(\,\cdot \,)^{\prime}$ denotes the first variation or partial derivative with respect to a design variable. The explicit design dependence terms of the strain and kinetic energy forms are given by

40 $${a^{\prime} }_{\delta {\bf{u}}}({\boldsymbol{\mu }};{\boldsymbol{\eta }},\bar{{\boldsymbol{\eta }}})\equiv {\int }_{{\rm{\Omega }}}({\bar{{\boldsymbol{\Gamma }}}}^{H}{{\bf{C}}{\boldsymbol{^{\prime} }}}_{F}{\boldsymbol{\Gamma }}+{\bar{{\boldsymbol{\Omega }}}}^{H}{{\bf{C}}{\boldsymbol{^{\prime} }}}_{M}{\boldsymbol{\Omega }})ds$$

and

41 $${d^{\prime} }_{\delta {\bf{u}}}({\boldsymbol{\mu }};{\boldsymbol{\eta }},\bar{{\boldsymbol{\eta }}})\equiv {\int }_{{\rm{\Omega }}}({\bar{{\bf{z}}}}^{H}\rho A^{\prime} {\bf{z}}+{\bar{{\boldsymbol{\theta }}}}^{H}{{\bf{I}}{\boldsymbol{^{\prime} }}}_{\rho }{\boldsymbol{\theta }})ds,$$

where the partial derivatives of the constitutive matrices and the cross-section properties can be evaluated using $\partial A/\partial {h}_{K}$ and $\partial {{\bf{I}}}_{\rho }/\partial {h}_{K}$, respectively. The partial derivative of the cross-sectional area and the inertial tensor with respect to *h*_*K*_ can be obtained, using the chain rule of differentiation and Eq. (38), as^34^

42 $$\frac{\partial A}{\partial {h}_{K}}=\frac{\partial A}{\partial h}{W}_{K}(\xi )$$

and

43 $$\frac{\partial {{\bf{I}}}_{\rho }}{\partial {h}_{K}}=\frac{\partial {{\bf{I}}}_{\rho }}{\partial h}{W}_{K}(\xi ).$$

### Harmonic response analysis

To investigate wave transmissions of finite size models constructed by tessellating primitive cells, we perform harmonic response analyses. Considering a harmonic external loading, we assume a harmonic response described as

44 $$\begin{array}{rcl}{\bf{z}}(s,t) & = & {\bf{z}}(s){e}^{-i{\rm{\Omega }}t}\\ {\boldsymbol{\theta }}(s,t) & = & {\boldsymbol{\theta }}(s){e}^{-i{\rm{\Omega }}t}\end{array}\},$$

where Ω is the excitation (angular) frequency. Then, we obtain^17^

45 $$({\bf{K}}+i{\rm{\Omega }}{\bf{C}}-{{\rm{\Omega }}}^{2}{\bf{M}}){\bf{u}}={\bf{f}},$$

where **f** denotes the applied harmonic force/moment load. The responses of dynamic system in Eq. (45) are computed by applying a given harmonic excitation at different values of frequencies (Ω). In this paper, the excitation is enforced by prescribing a displacement on a boundary point of the lattice, then collecting the magnitude of the response on the other side of the lattice. A damping matrix **C** is added to incorporate a structural damping, and it takes the form of Rayleigh (mass-proportional) damping such that

46 $${\bf{C}}=\beta {\bf{M}},$$

where *β* denotes the damping coefficient. The wave transmission coefficient (unit: dB) is calculated as

47 $$T=20\,{\mathrm{log}}_{10}(\frac{{U}_{out}}{{U}_{in}}),$$

where *U*_*in*_ and *U*_*out*_, respectively, denote the magnitude of input and output displacement vectors. We note that for the case of damped response, *U*_*in*_ and *U*_*out*_ of Eq. (47) are obtained by the magnitude of complex number components of the response u. This coefficient *T* quantifies the wave transmission at a specified frequency such that very low values of *T* means the applied perturbation quickly decays near the excitation point. A significant drop of *T* is associated with the band gap.

## Supplementary information

Supplementary information

CASE#1_2d_triangle

CASE#2_2d_triangle

CASE#3_2d_triangle

CASE#4_2d_hexagonal

CASE#5_2d_hexagonal

CASE#6_3d_square

CASE#7_2d_square

CASE#8_2d_kagome

CASE#9_2d_kagome
