# Supplementary material for: Optimal design of lattice structures for controllable extremal band gaps
Source: Sci Rep. 2019 Jul 10;9:9976. doi: 10.1038/s41598-019-46089-9 (PMC6620436; doi:10.1038/s41598-019-46089-9)
Supplement: Supplementary file 1 — Supplementary information [file 41598_2019_46089_MOESM1_ESM.doc]

**Supplementary information**

**TITLE:** Optimal design of lattice structures for controllable extremal band gaps

**AUTHOR NAMES:** Myung-Jin Choi^1^, Myung-Hoon Oh^1^, Bonyong Koo^2^, Seonho Cho^1*^

**AUTHOR ADDRESS:**

^1^Department of Naval Architecture and Ocean Engineering, Seoul National University,

1 Gwanak-ro, Gwanak-gu, Seoul 088826, Republic of Korea

^2^School of Mechanical Convergence System Engineering, Kunsan National University, Gunsan, Korea

^*^Corresponding authors’ E-mail address: [secho@snu.ac.kr](mailto:secho@snu.ac.kr)

***A****. Bloch theorem*

***B****. Direct and reciprocal lattice base vectors*

***C****. Square lattice structure*

***D****. Kagomé lattice structure*

***E****. Finite structures for harmonic response analyses*

*F. Description of video files*

# ***A. Bloch theorem***

A wave propagation in periodic lattice structures is investigated using a Bloch theorem, which enables to reduce the computation domain to single unit cell by taking advantage of periodicity. An infinite periodic lattice is described by a periodic arrangement of primitive unit cell through lattice base vectors $\mathbf{b}_{i} (i=1,2,3)$. The position **r**_p_ of any point *p* within the lattice is related to the corresponding position **r**_0_ in the unit cell as

$\mathbf{r}_{p}=\mathbf{r}_{0}+n_{1}\mathbf{b}_{1}+n_{2}\mathbf{b}_{2}+n_{3}\mathbf{b}_{3}$,

where $n_{i} (i=1,2,3)$ are integers. The Bloch theorem connects a response at any point *p* in the lattice to that of the corresponding point in the unit cell through the following equations,

$\left.\begin{matrix} \mathbf{z}(\mathbf{r}_{p},t)=\mathbf{z}(\mathbf{r}_{0},t)e^{\mathbf{k}\cdot(\mathbf{r}_{p}-\mathbf{r}_{0})}=\mathbf{z}(\mathbf{r}_{0},t)e^{n_{1}\mu_{1}+n_{2}\mu_{2}+n_{3}\mu_{3}} \\ \mathbf{\theta}(\mathbf{r}_{p},t)=\mathbf{\theta}(\mathbf{r}_{0},t)e^{\mathbf{k}\cdot(\mathbf{r}_{p}-\mathbf{r}_{0})}=\mathbf{\theta}(\mathbf{r}_{0},t)e^{n_{1}\mu_{1}+n_{2}\mu_{2}+n_{3}\mu_{3}} \end{matrix}\right\}$,

where $\mathbf{k}$ denotes the wave vector. The components of propagation constant $\mu_{i}=\mathbf{k}\cdot\mathbf{b}_{i}, (i=1,2,3)$ are complex numbers such that their real and imaginary parts represent the attenuation and phase constants, respectively. In this paper, we assume a purely propagating wave, $\mathrm{Re}(\mu_{i})=0$. To fulfill the periodicity of structural responses, the wave vector is expressed from Eq. (2), using reciprocal lattice bases $\mathbf{b}_{i}^{*},(i=1,2,3)$ that are defined to satisfy the relation $\mathbf{b}_{i}\cdot\mathbf{b}_{j}^{*}=2\pi\delta_{ij}$ where $\delta_{ij}$ denotes the kronecker-delta symbol as (Bayat, A. & Gaitanaros, S. Wave Directionality in Three-Dimensional Periodic Lattices. *J. Appl.* Mech*.* 85, 011004 (2018)

$\mathbf{b}_{1}^{*}=2\pi\frac{\mathbf{b}_{2}\times\mathbf{b}_{3}}{\mathbf{b}_{1}\cdot(\mathbf{b}_{2}\times\mathbf{b}_{3})}, \mathbf{b}_{2}^{*}=2\pi\frac{\mathbf{b}_{3}\times\mathbf{b}_{1}}{\mathbf{b}_{1}\cdot(\mathbf{b}_{2}\times\mathbf{b}_{3})}, \text{and} \mathbf{b}_{3}^{*}=2\pi\frac{\mathbf{b}_{1}\times\mathbf{b}_{2}}{\mathbf{b}_{1}\cdot(\mathbf{b}_{2}\times\mathbf{b}_{3})}$.

***B. Direct and reciprocal lattice base vectors***

Table S1 shows the lattice base vectors and the vertices of the irreducible Brillouin zones (IBZs) for each of the lattice topologies. A set of wave vector components on the IBZ perimeter is required for the band structure calculation, and we define $\varepsilon_{i}\equiv\mathrm{Im}(\mu_{i})$ for convenience. In this paper, 80 uniformly-spaced discrete points on each edge of the IBZ are selected, at which the eigenvalue problem is solved to calculate the band structures.

**Table S1.** Lattice base vectors of various honeycomb structures

| Lattice topology | Direct lattice vector | Reciprocal lattice vector | Irreducible Brillouin zone |
| --- | --- | --- | --- |
| Square | $\mathbf{b}_{1}=2L\mathbf{e}_{1}$ $\mathbf{b}_{2}=2L\mathbf{e}_{2}$ | $\mathbf{b}_{1}^{*}=\frac{\pi}{L}\mathbf{e}_{1}$ $\mathbf{b}_{2}^{*}=\frac{\pi}{L}\mathbf{e}_{2}$ | $\left.\begin{matrix} O:[\varepsilon_{1},\varepsilon_{2}]=[0,0] \\ A:[\varepsilon_{1},\varepsilon_{2}]=[\pi,0] \\ B:[\varepsilon_{1},\varepsilon_{2}]=[\pi,\pi] \end{matrix}\right\}$ |
| Triangular | $\mathbf{b}_{1}=L\mathbf{e}_{1}$ $\mathbf{b}_{2}=L\left(\frac{1}{2}\mathbf{e}_{1}+\frac{\sqrt{3}}{2}\mathbf{e}_{2}\right)$ | $\mathbf{b}_{1}^{*}=\frac{2\pi}{L}\left(\mathbf{e}_{1}-\frac{1}{\sqrt{3}}\mathbf{e}_{2}\right)$ $\mathbf{b}_{2}^{*}=\frac{2\pi}{L}\left(\frac{2}{\sqrt{3}}\mathbf{e}_{2}\right)$ | $\left.\begin{matrix} O:[\varepsilon_{1},\varepsilon_{2}]=[0,0] \\ A:[\varepsilon_{1},\varepsilon_{2}]=[0,\pi] \\ B:[\varepsilon_{1},\varepsilon_{2}]=[2\pi/3,4\pi/3] \end{matrix}\right\}$ |
| Hexagonal | $\mathbf{b}_{1}=\sqrt{3}L\left(\frac{1}{2}\mathbf{e}_{1}+\frac{\sqrt{3}}{2}\mathbf{e}_{2}\right)$ $\mathbf{b}_{2}=\sqrt{3}L\left(-\frac{1}{2}\mathbf{e}_{1}+\frac{\sqrt{3}}{2}\mathbf{e}_{2}\right)$ | $\mathbf{b}_{1}^{*}=\frac{2\pi}{\sqrt{3}L}\left(\mathbf{e}_{1}+\frac{1}{\sqrt{3}}\mathbf{e}_{2}\right)$ $\mathbf{b}_{2}^{*}=\frac{2\pi}{\sqrt{3}L}\left(-\mathbf{e}_{1}+\frac{1}{\sqrt{3}}\mathbf{e}_{2}\right)$ | $\left.\begin{matrix} O:[\varepsilon_{1},\varepsilon_{2}]=[0,0] \\ A:[\varepsilon_{1},\varepsilon_{2}]=[\pi,\pi] \\ B:[\varepsilon_{1},\varepsilon_{2}]=[4\pi/3,2\pi/3] \end{matrix}\right\}$ |
| Kagomé | $\mathbf{b}_{1}=L\left(\mathbf{e}_{1}+\sqrt{3}\mathbf{e}_{2}\right)$ $\mathbf{b}_{2}=L\left(-\mathbf{e}_{1}+\sqrt{3}\mathbf{e}_{2}\right)$ | $\mathbf{b}_{1}^{*}=\frac{\pi}{L}\left(\mathbf{e}_{1}+\frac{\sqrt{3}}{3}\mathbf{e}_{2}\right)$ $\mathbf{b}_{2}^{*}=\frac{\pi}{L}\left(-\mathbf{e}_{1}+\frac{\sqrt{3}}{3}\mathbf{e}_{2}\right)$ | $\left.\begin{matrix} O:[\varepsilon_{1},\varepsilon_{2}]=[0,0] \\ A:[\varepsilon_{1},\varepsilon_{2}]=[\pi,\pi] \\ B:[\varepsilon_{1},\varepsilon_{2}]=[4\pi/3,2\pi/3] \end{matrix}\right\}$ |
| Simple cubic | $\mathbf{b}_{1}=2L\mathbf{e}_{1}$ $\mathbf{b}_{2}=2L\mathbf{e}_{2}$ $\mathbf{b}_{3}=2L\mathbf{e}_{3}$ | $\mathbf{b}_{1}^{*}=\frac{\pi}{L}\mathbf{e}_{1}$ $\mathbf{b}_{2}^{*}=\frac{\pi}{L}\mathbf{e}_{2}$ $\mathbf{b}_{3}^{*}=\frac{\pi}{L}\mathbf{e}_{3}$ | $\left.\begin{matrix} O:[\varepsilon_{1},\varepsilon_{2},\varepsilon_{3}]=[0,0,0] \\ A:[\varepsilon_{1},\varepsilon_{2},\varepsilon_{3}]=[0,\pi,0] \\ B:[\varepsilon_{1},\varepsilon_{2},\varepsilon_{3}]=[\pi,\pi,0] \\ C:[\varepsilon_{1},\varepsilon_{2},\varepsilon_{3}]=[\pi,\pi,\pi] \end{matrix}\right\}$ |

***C. Square lattice structure***

We consider a planar square lattice structure composed of squares with the side length of 40*mm* on each side. Considering the translational periodicity in *X*- and *Y*-directions, the direct lattice base vectors $\mathbf{b}_{1}$, $\mathbf{b}_{2}$, and the unit cell is defined, as illustrated in Figure S1(a). We select a rectangular cross-section with uniform thickness $h_{0}=2mm$ and depth $d_{0}=6mm$, material properties of Young’s modulus $E=1.14GPa$, Poisson’s ratio $\nu=0.3$, and mass density $\rho=1050kg/m^{3}$. Also, Fig. S1(a) shows the reciprocal lattice bases $\mathbf{b}_{1}^{*}$ and $\mathbf{b}_{2}^{*}$ whose detailed expressions are found in *Supplement B*, from which we identify the irreducible Brillouin zone whose boundary O-A-B. Fig. S1(b) illustrates the parameterization of configuration design variables. Within a quarter part of unit cell, the configuration of one ligament is parameterized by 6 control points in a coarse level discretization using quartic B-spline basis functions, and the other 3 ligaments are parameterized by using a rotational symmetry. 14 thickness control coefficients are used for thickness design parameterization of each ligament, i.e., $n_{th}=14$. For the straight design in Fig. S1(c), no complete band gap appears in Fig. S1(d). When we introduce an undulated geometry shown in Fig. S1(c) by perturbing design variables such as $d_{i}=5mm (i=1\sim4)$, a couple of complete band gaps appear in Fig. S1(e).

**
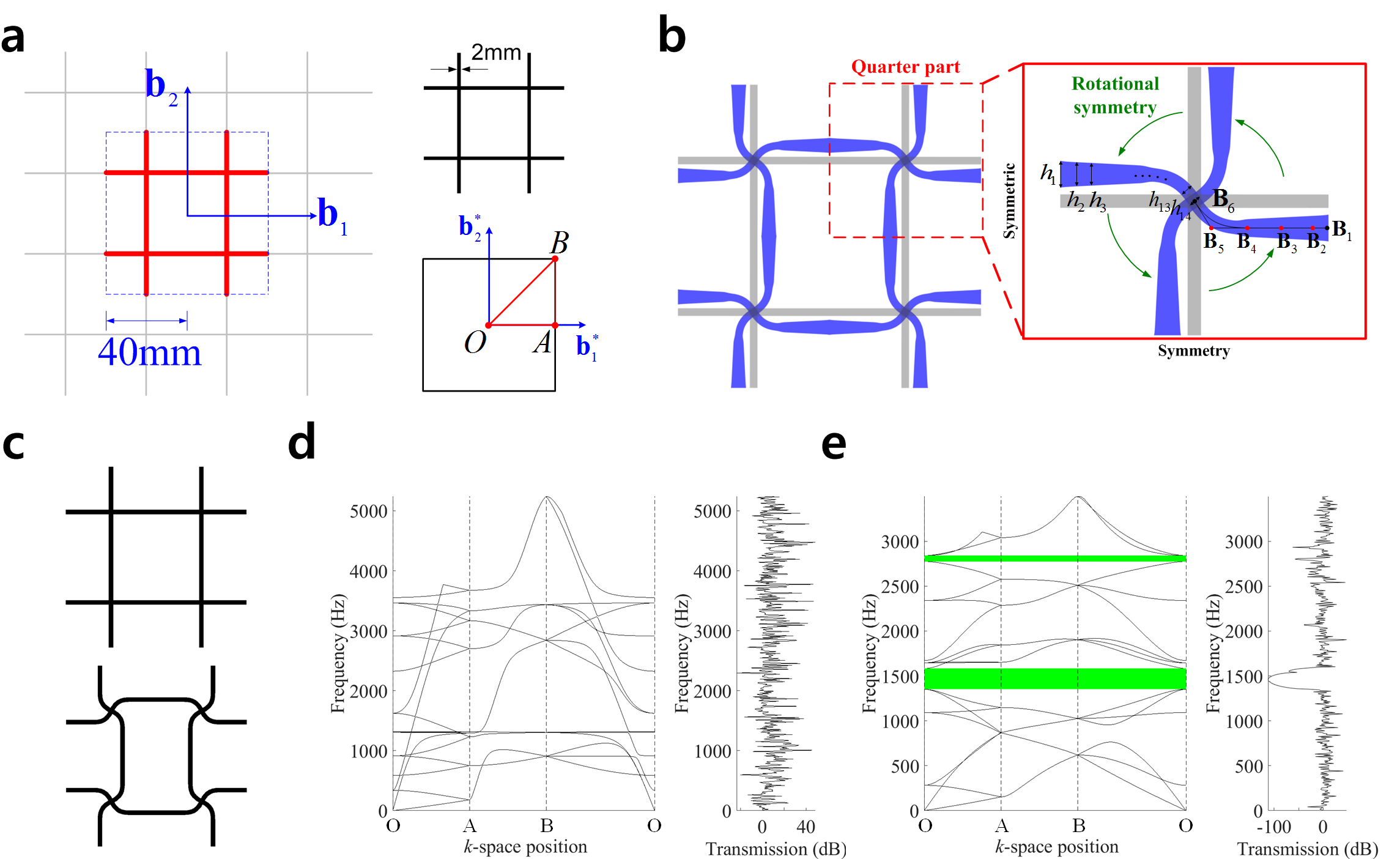
**

**Figure S1.** Square lattice structure: (a) Unit cell and irreducible first Brillouin zone,
(b) Design parameterization, (c) Straight and undulated structures, (d) Band structures and frequency responses of straight structures (e) Band structures and frequency responses of undulated structures

The band gap between 8^th^ and 9^th^ modes is selected to be maximized by the optimization process (Case #7). Fig. S2(a) shows the optimization results. The decrease of cross-section thickness around the junction is noticeable, and the other parts become thicker than those of the original design. Fig. S2(b) shows that a very large band gap is generated between the target 8^th^ and 9^th^ modes at the frequency range of 553~2,762Hz where the wave transmission is shown to be significantly suppressed. Table S2 compares the band gap sizes and the frequency ranges of the original undulated and the optimal designs. Fig. S2(c) shows the history of objective function during the optimization, where the optimal design is sought through only a few iterations.


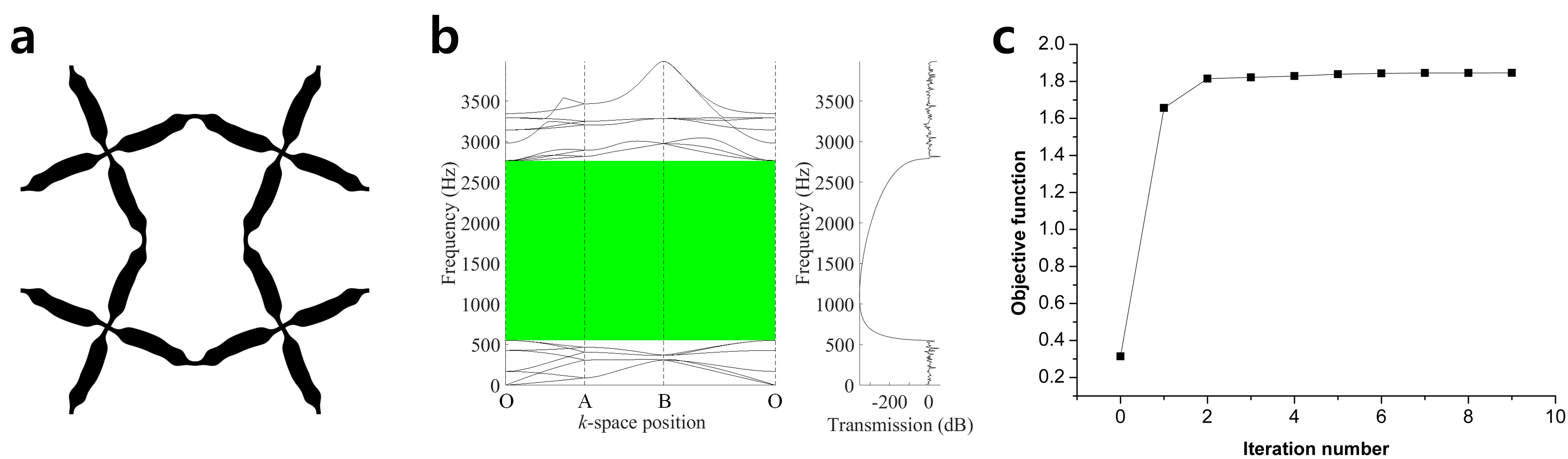


**Figure S2.** Optimal undulated design of square lattice (a) Unit cell (b) Band structures and frequency responses, (c) Objective function history for square lattice structure

**Table S2.** Comparison of band gap sizes and frequency ranges

|  | Band gap # | Band gap size | Lower bound of band gap | |
| --- | --- | --- | --- | --- |
|  |  |  | Mode# | Frequency (Hz) |
| Original undulated design | 1 | 231.8 | 8 | 1350.9 |
|  | 2 | 65.8 | 16 | 2774.4 |
| Optimal design | 1 | 2209.2 | 8 | 552.8 |

***D. Kagomé lattice structure***

We consider a planar Kagomé lattice structure composed of regular triangles with alternating orientations and each side of 40*mm* long, as illustrated in Fig. S3(a). Due to the translational periodicity in the direction of base vectors $\mathbf{b}_{1}$ and $\mathbf{b}_{2}$, a unit cell is defined in Fig. S3(b). We select a rectangular cross-section with uniform thickness $b_{0}=2mm$ and depth $d_{0}=6mm$, and material properties of Young’s modulus $E=1.14GPa$, Poisson’s ratio $\nu=0.3$, and mass density $\rho=1050kg/m^{3}$. Fig. S3(c) shows the reciprocal lattice bases $\mathbf{b}_{1}^{*}$ and $\mathbf{b}_{2}^{*}$ whose detailed expression can be found in *Supplement B*, from which an irreducible Brillouin zone (boundary O-A-B) is determined.


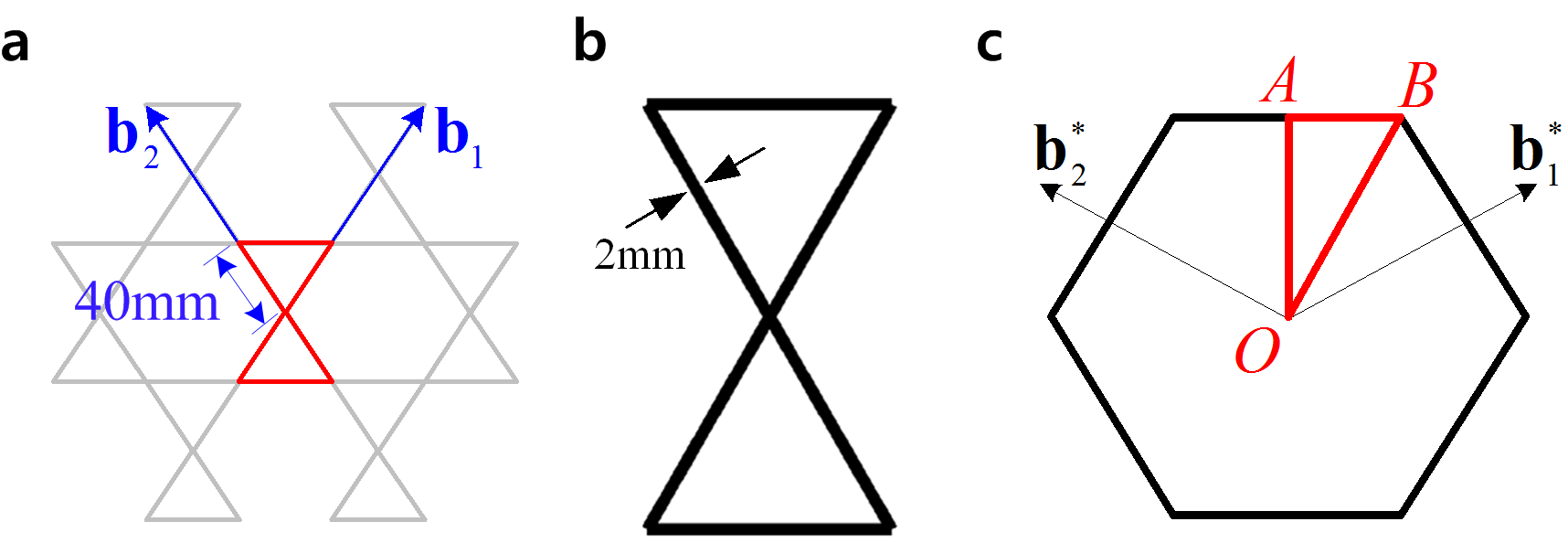


**Figure S3.** Kagomé structure: (a) Kagomé lattice, (b) Unit cell, (c) Irreducible Brillouin zone

Fig. S4(a) shows that this structure has no complete band gap. A single ligament configuration within the half part of the unit cell is parameterized by 8 configuration design variables in a coarse level discretization by quartic B-spline basis functions, and the other ligaments are parameterized by exploiting a rotational symmetry within the half part and the point symmetry within the unit cell, as illustrated in Fig. S4(b). 14 cross-section thickness control coefficients are used in each ligament, i.e., $n_{th}=14$.


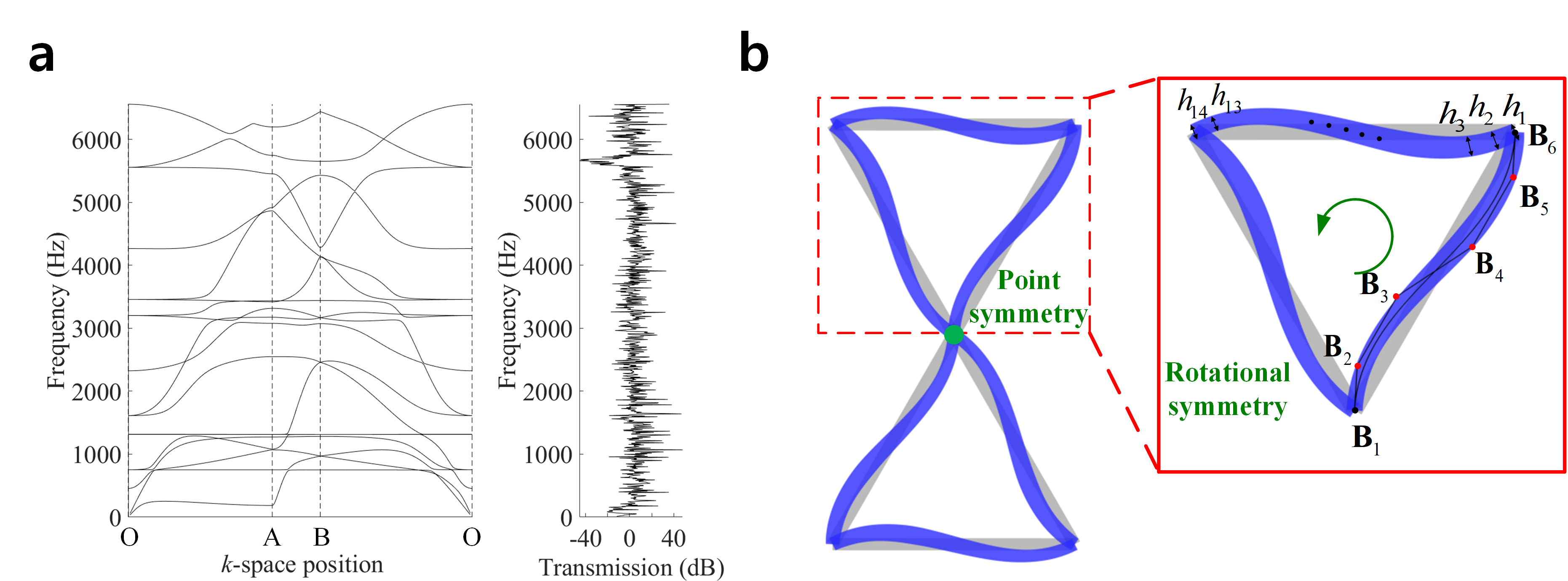


**Figure S4.** (a) Band structures and frequency responses of original straight design,
(b) 8 configuration design variables for Kagomé lattice

We introduce an undulated design of Fig. S5(a) by perturbing the design variables as $d_{1}=d_{2}=2.5mm$ and $d_{1}=d_{2}=-2.5mm$. This undulated design shows a couple of complete band gaps in Fig. S5(b). From this undulated design, we perform two design optimizations for maximizing target band gaps between 9^th^-10^th^ modes (Case #8) and 3^rd^-4^th^ modes (Case #9), respectively. In the design optimization of case #8, we employ the following additional constraints imposing a minimum distance between selected discrete points on neutral axes to reduce overlap of *m*-th and *n*-th ligaments around a junction. $n_{d}$ denotes the number of discrete points selected in each ligament^26^.

, $i,j=1,...,n_{d}$,

where $D_{mn}$ and $D_{mn}^{0}$ denote a distance between selected points and a specified minimum distance, respectively. Figs. S6 and S7 show the optimization results, and significantly large band gaps at low frequency levels are noticeable, and the wave transmissions are also much lower at those frequency regions, compared with those of the original undulated design. Table S3 compares the band gap sizes and frequency ranges of the original undulated design and two cases of optimal designs. Both of the optimum solutions converge after about 10 iterations as show in Fig. S8. The initial decrease of objective function in the case #8 occurs during the design change in the direction to feasible region of the constraints of Eq. .


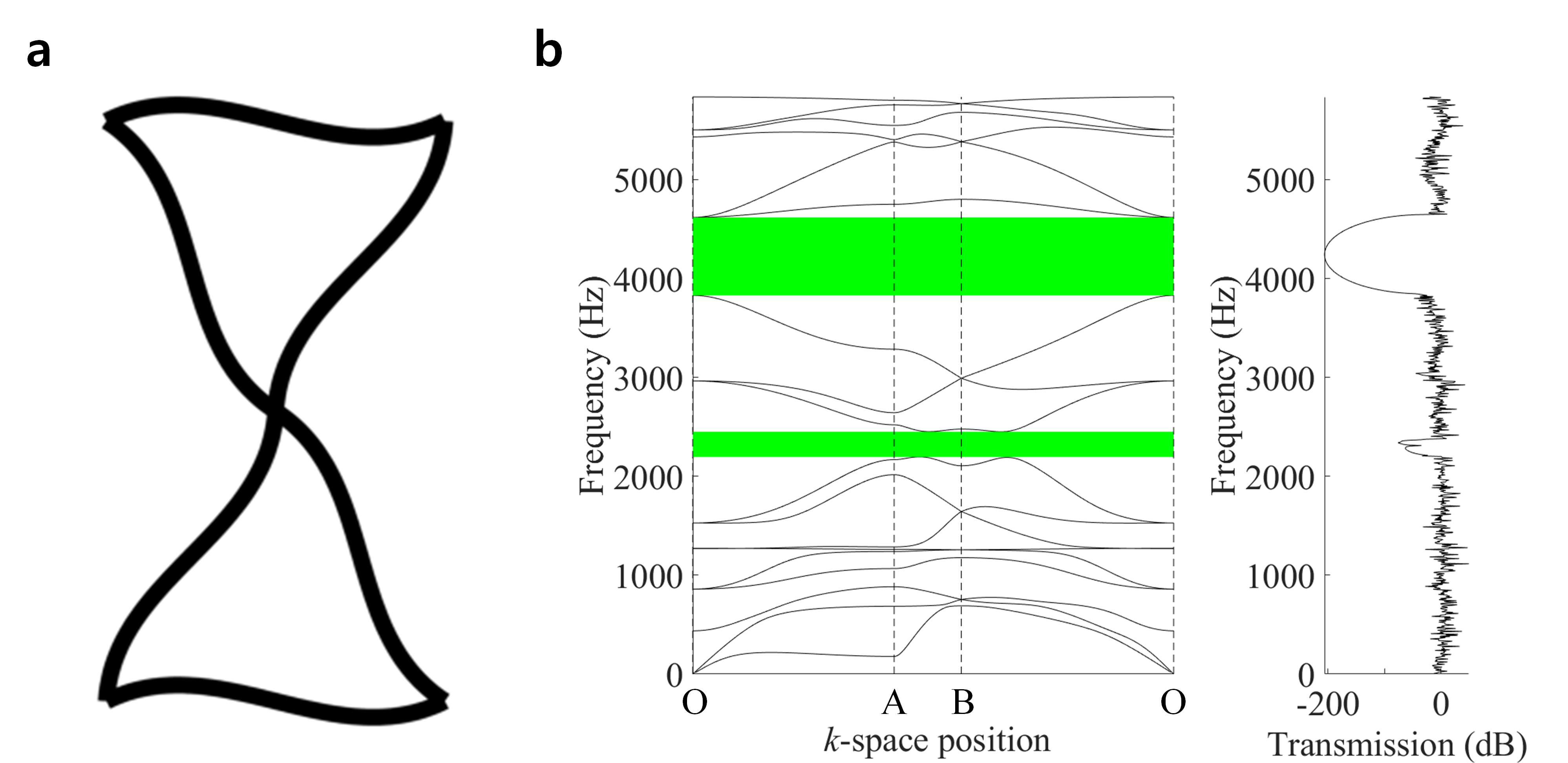


**Figure S5.** Wave attenuation properties of original undulated design of Kagomé lattice:
(a) Original design, (b) Band structures and frequency responses of original undulated design


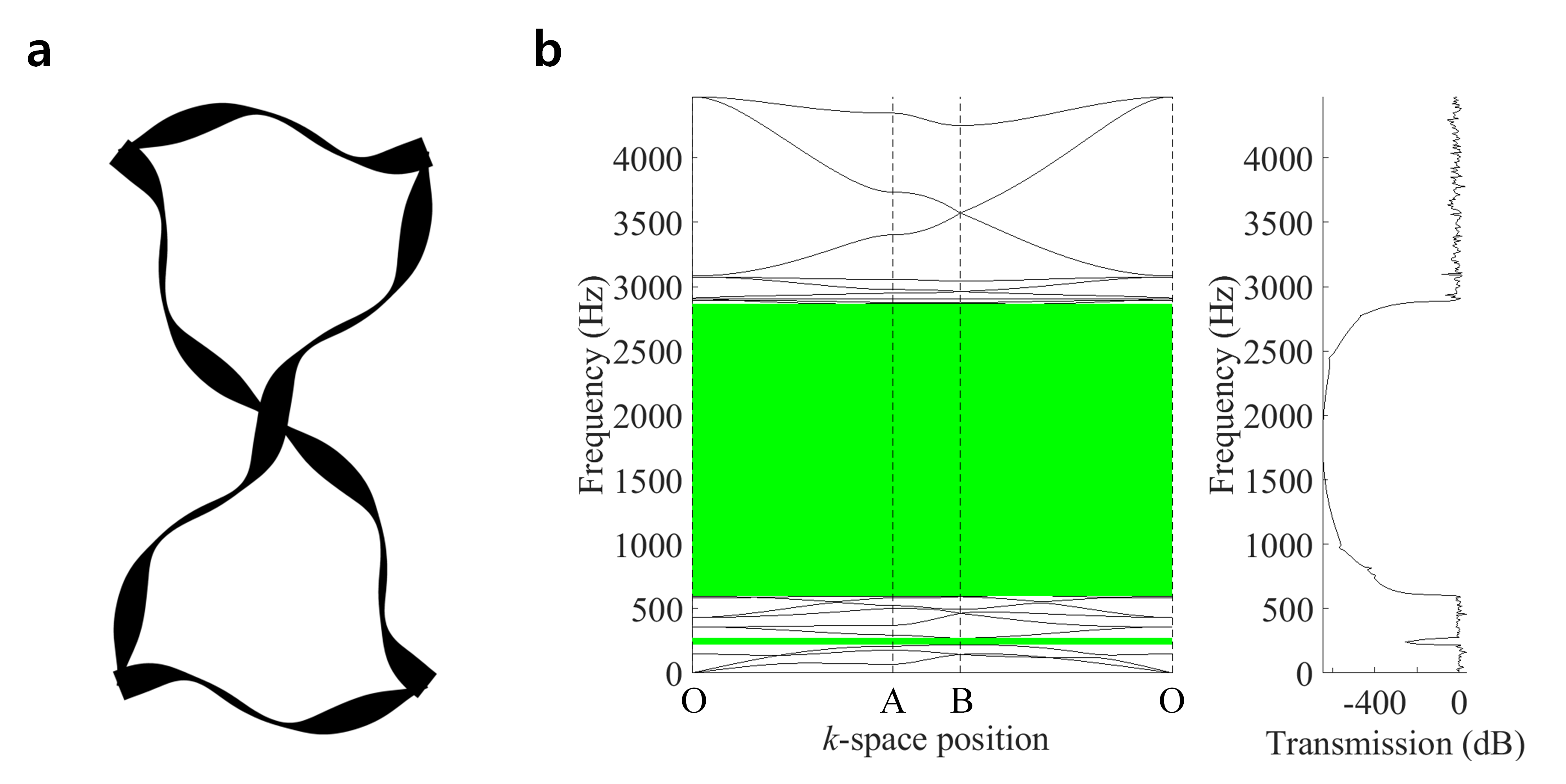


**Figure S6.** Case #8 optimal design of Kagomé lattice (a) Optimal design for target band gap between 9^th^ and 10^th^ modes (b) Band structure and frequency response


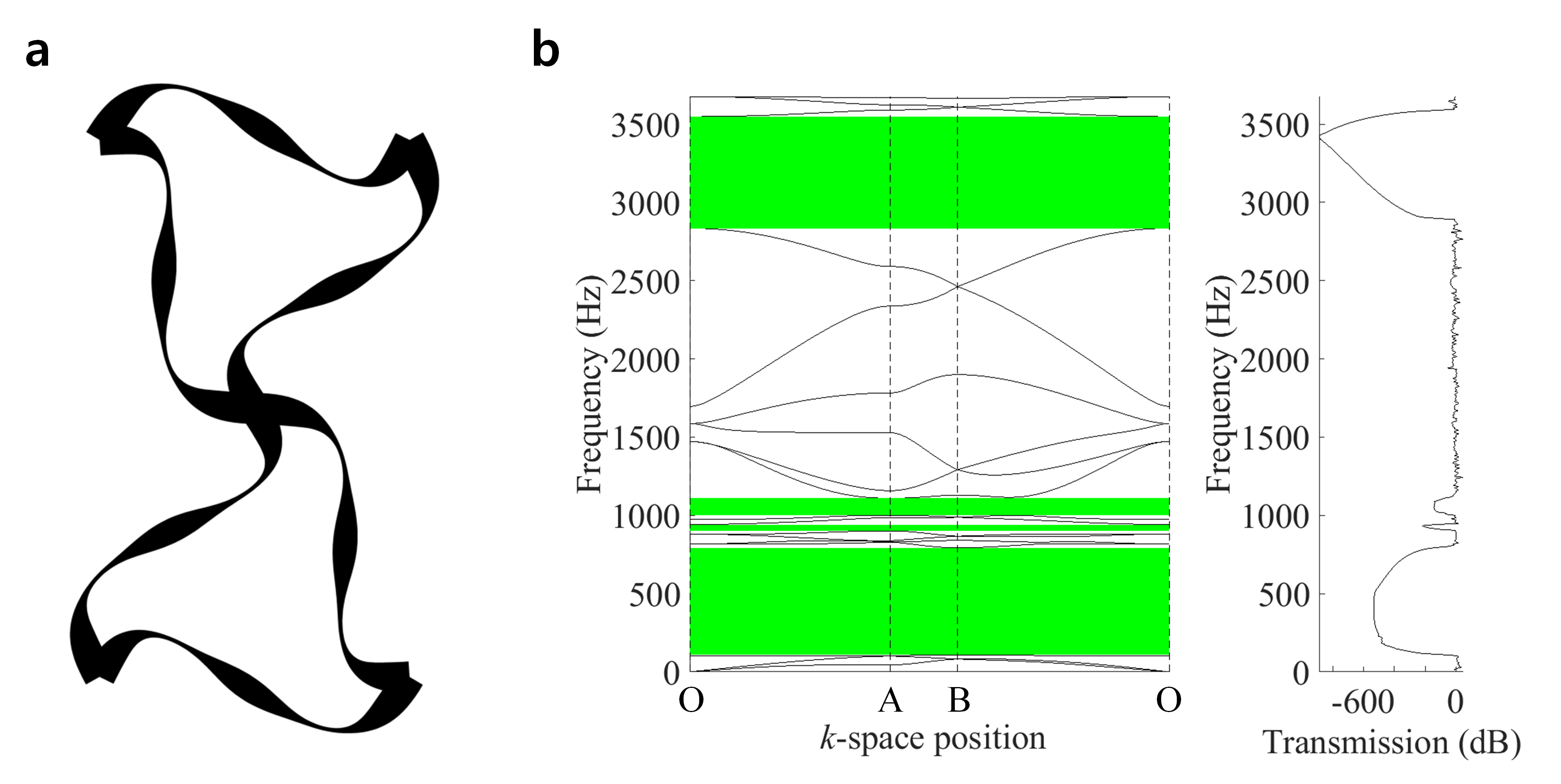


**Figure S7.** Case #9 optimal design of Kagomé lattice (a) Optimal design for target band gap between 3^rd^ and 4^th^ modes (b) Band diagram and frequency response


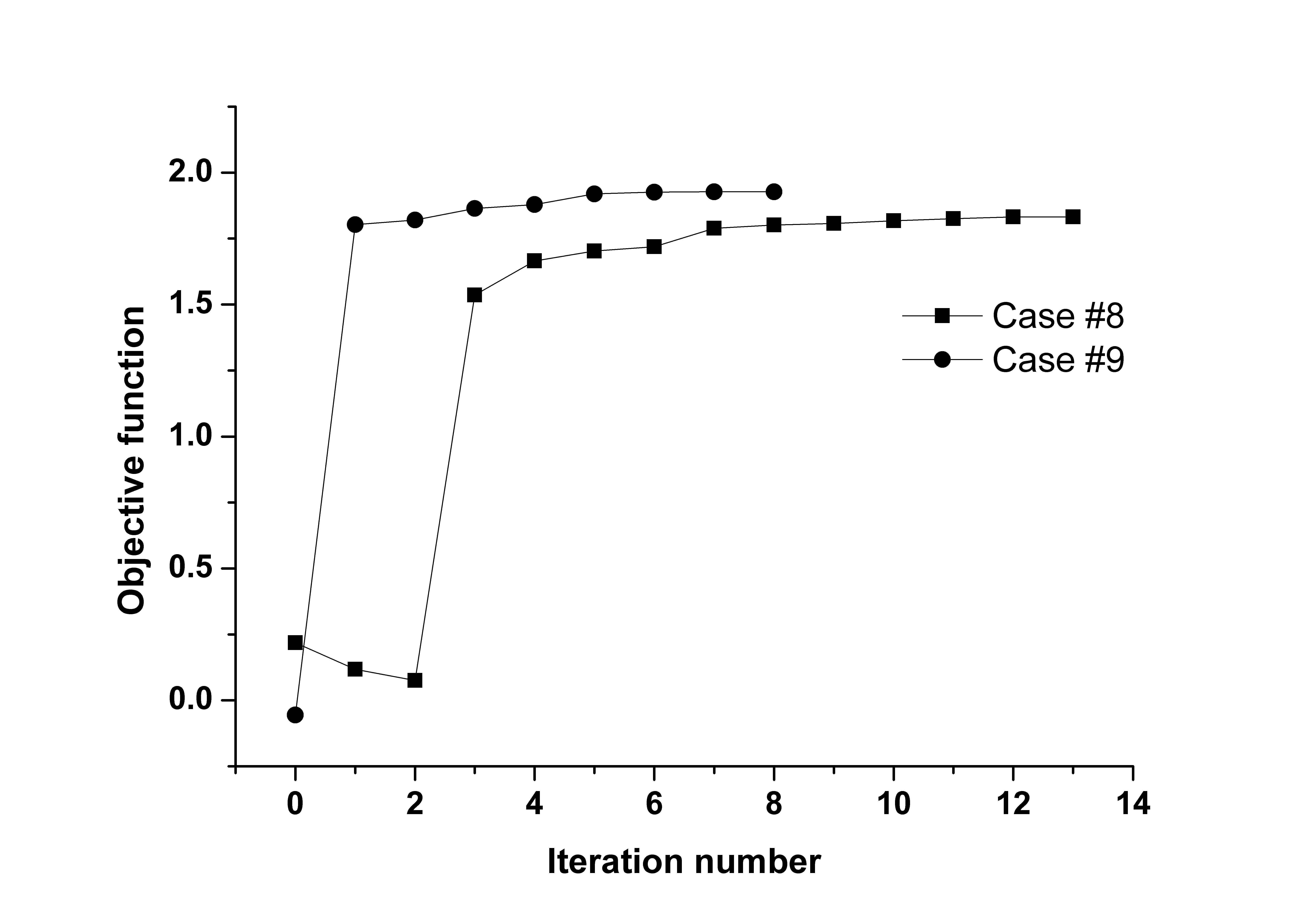


**Figure S8.** Objective function history for kagomé lattice structure

**Table S3.** Comparison of band gap sizes and frequency ranges

|  | Band gap # | Band gap size  (Hz) | Lower bound of band gap | |
| --- | --- | --- | --- | --- |
|  |  |  | Mode # | Frequency (Hz) |
| Original undulated design | 1 | 254.1 | 9 | 2196.5 |
|  | 2 | 788.8 | 12 | 3829.5 |
| Optimal design (case#8) | 1 | 53.0 | 3 | 218.5 |
|  | 2 | 2265.7 | 9 | 600.2 |
| Optimal design (case#9) | 1 | 685.1 | 3 | 107.7 |
|  | 2 | 40.2 | 7 | 901.3 |
|  | 3 | 109.3 | 9 | 1000.5 |
|  | 4 | 713.0 | 15 | 2834.8 |

***E. Finite structures for harmonic response analyses***

We present finite size structural models constructed by assembling unit cells for the harmonic response analyses. Fig. S9 shows the two-dimensional finite structures for each of the lattice structural models, where straight geometry models are illustrated, and the excitation and measurement positions are indicated. Here, $n_{1}\times n_{2}\times n_{3}$ array means $n_{i}$ unit cells are arranged in the direction of base vector $\mathbf{b}_{i} (i=1,2,3)$. Fig. S10 shows the finite structures for the three-dimensional simple cubic lattice model with straight geometry, where the excitation is enforced at the mid-point of the upper boundary, and the response is measured at the other side. The undulated design and optimal design models employ the same array of unit cells as the corresponding straight structures.


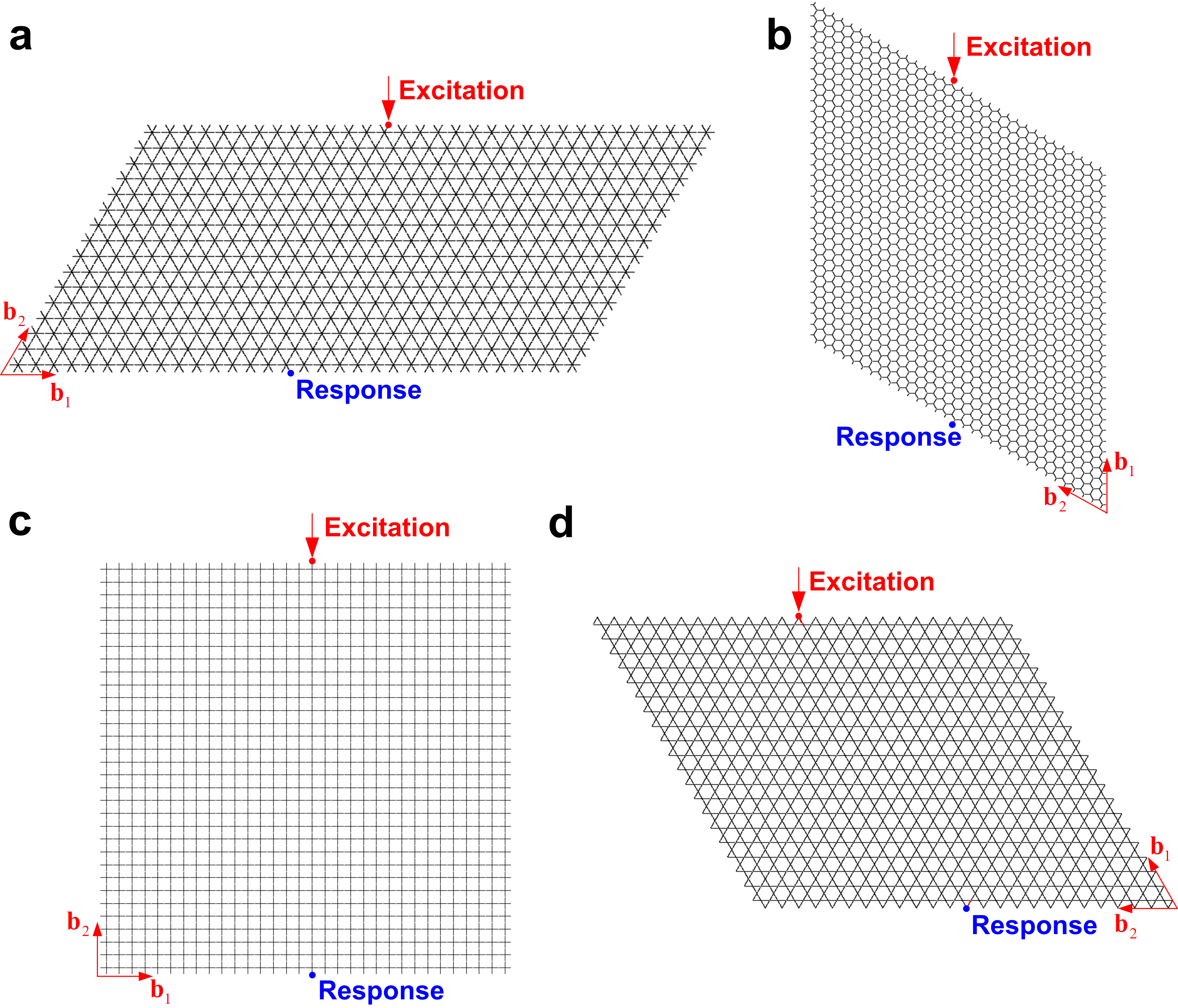


**Figure S9.** Two-dimensional finite structures used in the harmonic response analyses
(a) Triangular lattice with 32×16 array of unit cells (b) Hexagonal lattice with 32×32 array
(c) Square lattice with 16×16 array (d) Kagomé lattice with 20×25 array


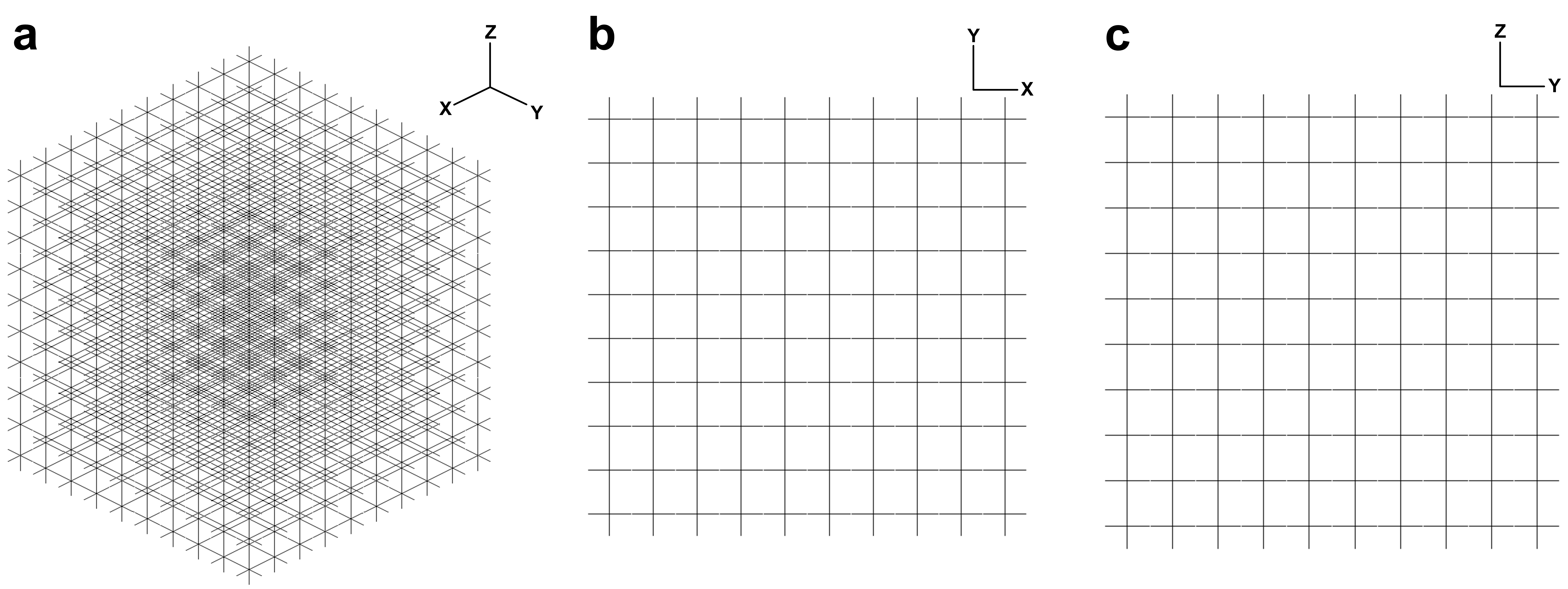


**Figure S10.** A finite structure by 10×10×10 array of simple cubic unit cells
(a) Perspective view (b) XY-plane view (c) YZ-plane view

***F. Description of video files***

The following video files show the history of configuration and sizing design optimization.

**(1) CASE#1_2d_triangle:**

Straight beams. Target band gap is between 3^rd^ and 4^th^ modes.

**(2) CASE#2_2d_triangle:**

Undulated beams. Target band gap is between 3^rd^ and 4^th^ modes.

**(3)** **CASE#3_2d_triangle:**

Undulated beams. Target band gap is between 6^th^ and 7^th^ modes.

**(4)** **CASE#4_2d_hexagonal:**

Undulated beams. Target band gap is between 3^rd^ and 4^th^ modes.

**(5)** **CASE#5_2d_hexagonal:**

Undulated beams. Target band gap is between 9^th^ and 10^th^ modes.

**(6)** **CASE#6_3d_square:**

3-dimensional undulated beams. Target band gap is between 15^th^ and 16^th^ modes.

**(7)** **CASE#7_2d_square:**

Undulated beams. Target band gap is between 8^th^ and 9^th^ modes.

**(8)** **CASE#8_2d_kagome:**

Undulated beams. Target band gap is between 9^th^ and 10^th^ modes.

**(9)** **CASE#9_2d_kagome:**

Undulated beams. Target band gap is between 3^rd^ and 4^th^ modes.
